# ER Negative Breast Cancer and miRNA: There Is More to Decipher Than What the Pathologist Can See!

**DOI:** 10.3390/biomedicines11082300

**Published:** 2023-08-18

**Authors:** Ghada Chamandi, Layal El-Hajjar, Abdallah El Kurdi, Morgane Le Bras, Rihab Nasr, Jacqueline Lehmann-Che

**Affiliations:** 1 Department of Anatomy, Cell Biology and Physiological Sciences, Faculty of Medicine, American University of Beirut, 11-0236 Beirut, Lebanon; gc21@aub.edu.lb (G.C.); lh85@aub.edu.lb (L.E.-H.); 2 Pathophysiology of Breast Cancer Team, INSERM U976, Immunologie Humaine, Pathophysiologie, Immunothérapie (HIPI), Université Paris Cité, 75010 Paris, France; morgane.le-bras@u-paris.fr; 3 Office of Basic/Translational Research and Graduate Studies, Faculty of Medicine, American University of Beirut, 11-0236 Beirut, Lebanon; 4 Department of Biochemistry and Molecular Genetics, Faculty of Medicine, American University of Beirut, 11-0236 Beirut, Lebanon; ak161@aub.edu.lb

**Keywords:** breast cancer, triple negative breast cancer, molecular apocrine breast cancer, luminal androgen breast cancer, biomarker, microRNA, androgen receptor

## Abstract

Breast cancer (BC), the most prevalent cancer in women, is a heterogenous disease. Despite advancements in BC diagnosis, prognosis, and therapeutics, survival rates have drastically decreased in the metastatic setting. Therefore, BC still remains a medical challenge. The evolution of high-throughput technology has highlighted gaps in the classification system of BCs. Of particular interest is the notorious triple negative BC, which was recounted as being heterogenous itself and it overlaps with distinct subtypes, namely molecular apocrine (MA) and luminal androgen (LAR) BCs. These subtypes are, even today, still misdiagnosed and poorly treated. As such, researchers and clinicians have been looking for ways through which to refine BC classification in order to properly understand the initiation, development, progression, and the responses to the treatment of BCs. One tool is biomarkers and, specifically, microRNA (miRNA), which are highly reported as associated with BC carcinogenesis. In this review, the diverse roles of miRNA in estrogen receptor negative (ER−) and androgen receptor positive (AR+) BC are depicted. While highlighting their oncogenic and tumor suppressor functions in tumor progression, we will discuss their diagnostic, prognostic, and predictive biomarker potentials, as well as their drug sensitivity/resistance activity. The association of several miRNAs in the KEGG-reported pathways that are related to ER-BC carcinogenesis is presented. The identification and verification of accurate miRNA panels is a cornerstone for tackling BC classification setbacks, as is also the deciphering of the carcinogenesis regulators of ER − AR + BC.

## 1. Introduction

Breast cancer (BC) is depicted as the most common cancer in women, with an estimated number of 2.3 million new cases worldwide in 2020 [1]. This incidence is predicted to increase in the next 15 years due to cancer screening tests, but also because of growing risk factors like increases in excess body weight [2,3]. A recent analysis of United States (US) cancer data, by The American Society of Cancer, revealed a slow increase in BC incidence (0.5% per year) since the mid-2010s. In parallel, for 30 years, female BC mortality has decreased, and this is mainly because of earlier diagnoses and improved treatments; however, this effect has been slowing in the last few years. Thus, BC remained as among the first causes of worldwide cancer deaths in 2020 [1], with 43.2 thousand estimated deaths in the US for 2022 [4]. Although the 5-year relative survival rate of BC is 90%—constituting one of the best for prognostic cancers—late recurrences are frequent, and the survival rate decreases dramatically in the metastatic setting [5].

All of these facts highlight the fact that BC remains a medical challenge, and that it will continue to be one of the major health challenges in future years.

## 2. Breast Cancer Is a Highly Heterogeneous Disease

One of the main issues concerning BC is the high heterogeneity of the disease. Indeed, BC includes a vast array of histological and molecular subtypes [6,7] with clinical implications.

First, from a histological point of view, the large majority (70–80%) of invasive breast neoplasms occur through the infiltration of ductal carcinomas of no special type (IDC-NST), which is followed by invasive lobular carcinomas (8–15%) [8,9]. Other histologic types exist but are less common, and these include micropapillary, papillary, metaplastic, and apocrine carcinomas.

**Figure 1:**
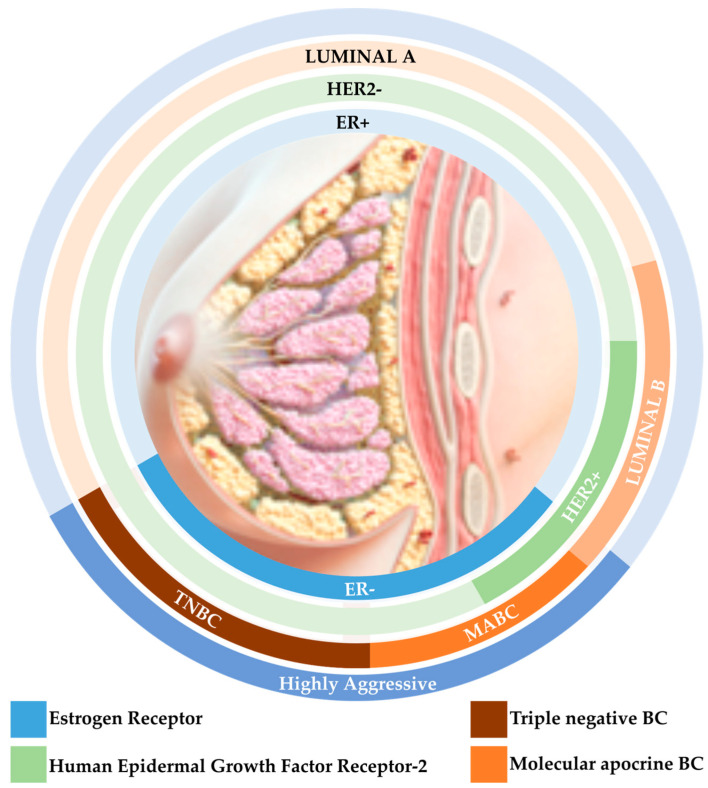
BC subtypes with respect to the receptors’ expression and correlation to aggressiveness.

Second, at a molecular level, a variety of subtypes have been described since 2000 with high therapeutic implications. Indeed, the advances in high-throughput technologies has allowed for a better biological demonstration of the BC heterogeneity at the molecular level, raising five intrinsic subtypes, which are hierarchically clustered into luminal A, luminal B, HER2-overexpressing, basal-like, and normal-like BCs [10]. Since this first transcriptomic molecular portrait of BC, multiple histopathological and biological features have been described for the purpose of a better classification and comprehension of the breast neoplasm, and this development has continued to evolve. However, four coherent groups can recurrently be defined by gene expression profiling [11]. This could be conducted possibly by multiparameter molecular tests such as PAM50 and, as is more often the case, with surrogate approaches such as by immunohistochemistry analysis. According to the St. Gallen 2013 consensus, BC molecular subtypes are defined according to estrogen receptors (ERs), progesterone receptors (PRs), Human Epidermal Growth Factor Receptor-2 (HER2), and the proliferation marker Ki67 expression as per the following: luminal A-like (ER+/PR+, HER2−, Ki67+ < 20%); luminal B-like HER2− (ER+/PR+ < 20%, HER2−, Ki67+ ≥ 20%); luminal B-like HER2+ (ER+/PR+, HER2 overexpression); HER2 overexpressed (non-luminal (ER−, PR−); HER2 overexpression); and basal-like and/or triple-negative BC (TNBC) (ER−, PR−, HER2−) [12] (Figure 1).

The luminal A-like tumors have clear prognostic and treatment implications as they proliferate less and are endocrine sensitive, thus it confers better prognosis but have a poor response to chemotherapy [13]. Luminal B-like tumors are of a higher Ki67 expression and grade, and they have less endocrine sensitivity and poorer prognoses [13,14]. HER2 overexpression leads to bad prognosis but also to a better prediction of the response to anti-HER2 therapies, which drastically improves patient survival. However, the non-luminal HER2+ group is fast growing, more aggressive, and presents a worse prognosis than luminal groups [15]. Finally, TNBCs—which account for 20% of BCs and is defined by the absence of the three major receptors of ER, PR, and HER2—present with an aggressive behavior that have a high proliferation and the most pejorative survival rates [13]. Moreover, as defined by what they are not, TNBCs remain a highly heterogeneous subgroup that need to be better characterized.

Importantly, the accurate definition of BC is necessary for proper diagnoses and treatment strategies. The huge heterogeneity of this disease is described in the WHO tumor classification [16], which was updated in 2019 [11].

## 3. Triple Negative Breast Cancers: What Are They?

TNBCs are characterized by clinical and pathological differences, as well as by distinct molecular expression profiles that translate into distinct behaviors and responses to chemotherapy. In general, TNBCs exert higher risks of recurrence with the emergence of brain and lung metastases that occur more frequently than bone metastasis when compared to other breast subtypes. Also, TNBC metastatic diseases appears rapidly within the first 3 years after diagnosis, thus leading to bad prognosis. However, when patients do not recur during this time, the survival rate is comparable to ER+ BC. Moreover, 30–40% of TNBC patients experience a pathological complete response (pCR) after neoadjuvant chemotherapy, and this constitutes a strong surrogate marker for overall survival. Therefore, it is clear that TNBCs are not a single clinico-pathological entity, but they need a better characterization of their more homogenous entities for the optimization of treatment.

Several gene expression studies have tried to dissect this heterogeneous group [17,18,19]. Initially, Lehmann et al. described six subgroups of TNBCs: basal-like 1 (BL1), basal-like 2 (BL2), immunomodulatory (IM), mesenchymal-(M), mesenchymal stem-like (MSL) and luminal androgen receptor (LAR) [17,20]. Finally, after the removal of immunological and stromal expression signals, this classification was refined into four tumor-specific subtypes (TNBCtype-4): BL1, BL2, M, and LAR. These subtypes have clear differences in their responses to chemotherapy [20]. Nevertheless, this subtyping is not currently used in routine practice. Moreover, the LAR subtype, with luminal characteristics but androgen receptor (AR) overexpression, should certainly be considered differently. In addition, the 2019 WHO classification recognized the existence of an ER− subtype, but AR+ mammary carcinoma was categorized as a distinct type of BC [7].

## 4. Apocrine Carcinoma: Just a Histology or a Molecular Entity?

Historically, breast apocrine carcinomas were defined by their particular morphological and histological appearances, with their tumor cells possibly presenting abundant granular cytoplasm, central nuclei positions, prominent nucleoli, and gross cystic disease fluid protein-15 (GCDFP-15) positive expressions by IHC [21,22]. This particular histology is also described in rare malignant adnexal neoplasms, which most commonly arise in areas with high-apocrine-gland densities, such as the axilla.

In 2005, after the transcriptomic profiling of BC, Farmer et al. described a new subtype of BC that is characterized by a luminal expression profile without ER but AR overexpression, as well as with a morphological apocrine differentiation (which was designated by the term molecular apocrine breast cancer (MABC) [23]). Subsequently, different groups have identified the MABC in non-redundant BC datasets [24]; these MABC tumors were recurrently found to specifically overexpress the AR gene and its consecutive pathway in an ER negative context with frequent expression/amplification of HER2 [23,24]. This led to the proposal of a new BC classification by Guedj et al., who split the HER2-like subtype of Perou and Sorlie into luminal B and MABC [25]. In parallel, Lehmann et al. published the TNBC subclassification described above and defined the LAR subtype as ER−/HER2−/AR+ [17,20]. Some confusion could be induced by these different descriptions, but it can be assumed that LARs probably converge on the HER2− part of the initially described MABC [25,26] (even if this has yet to be formally proven). Altogether, these data recently contributed to the consideration of these invasive MABC/LAR carcinomas as a subgroup of its own [27], leading to its inclusion in the WHO categorization of BC. This individualization of a subtype makes sense if distinct diagnoses, prognoses, or treatments are allowed by its identification as such.

## 5. MABC/LAR: How, and Why Are they Not Identified in Routine Practice?

MABC/LAR definition is based on the gene signatures obtained by messenger RNA (mRNA) expression profiling when they are not routinely performed. Some groups, including ours, have proposed MABC mRNA signatures or surrogate immunohistochemistry (IHC) markers as they are easier to apply [24,26]. However, currently, MABC/LAR profiling is not yet systematically performed.

Nevertheless, MABC/LARs are characterized by AR overexpression, and this can be easily evaluated by pathologists. Thus, MABC/LARs are essentially characterized by AR positive IHC in the context of an absence of ER and PR expressions. AR is a member of the sex steroid hormone receptor family (like ER, PR, etc.), and it is expressed in several human tissues including the breast [28]. In the context of BC, AR is overexpressed in more than 70% of cases, so it represents the greatest largely expressed hormone receptor [29]. However, it seems clear that AR plays a different role if associated with the presence or absence of ER overexpression [30].

In the ER− MABC/LAR context, the proof of concept and clinical trials supporting the targeting of AR by anti-AR drugs has come away with modest and controversial results [31,32,33,34,35,36,37,38]. Some inconsistencies could be explained by the lack of standardized AR evaluation, which is an obstacle that constitutes a major limitation for the proper definition of the subtype. Indeed, no consensus exists for the use of specific anti-AR antibodies, protocols, and positive cut-off scores. Moreover, the comparison of AR IHC evaluation and mRNA MABC signatures has demonstrated a weak concordance between these two classification tools [26]. Finally, the identification of this subtype remains a challenge, and better means for identifying it are hence needed to refine its diagnosis, prognosis, and treatment. With respect to novel and potentially useful biomarkers, microRNA (miRNA) appears to be a promising diagnostic biomarker. Moreover, the miRNA network could also help to better define the carcinogenesis of MABC/LARs and their behavior. Accordingly, in this review, we will focus on the potential role of specific dysregulated miRNA profiles in TNBC. More interestingly (in that of the less known ER−AR+ subtypes), we will also explore new approaches in order to understand and diagnose MABC/LAR breast tumors.

## 6. Search Strategy

A search strategy was adopted for the following part of the study, and two approaches were applied. The miRNAs in TNBCs were targeted by using the PubMed medical subject heading (MeSH) database. PubMed was searched for the following: “Breast Neoplasms” [MeSH] AND “MicroRNA” [MeSH] AND biomarkers AND prognosis AND diagnosis. For miRNA-AR interaction, the following terms were searched: “MicroRNA”[MeSH Terms] AND (“receptors, androgen”[MeSH Terms] OR (“receptors”[All Fields] AND “androgen”[All Fields]) OR “androgen receptors”[All Fields] OR (“androgen”[All Fields] AND “receptors”[All Fields])) AND (“breast neoplasms”[MeSH Terms].

## 7. microRNA

miRNAs are small non-coding RNAs of about 18–25 nucleotides in length. Most of these miRNAs bind to the 3′ untranslated regions of target mRNAs, thus regulating gene expression at the post-transcriptional level and leading to mRNA cleavage, translational suppression, or deadenylation [39,40,41]. In humans, it is estimated that almost a third of mRNAs are controlled by miRNAs. In fact, this is a complex network of interactions where one miRNA may bind to as much as 200 targets, and a single gene can be regulated by various miRNAs [42,43]. Rarely does a miRNA activate mRNA translation and elevate target protein levels [44]. The miRNA-mediated regulation of gene expression was highlighted by a number of studies that revealed that miRNAs play a pivotal role in physiological and pathological processes [45,46]. miRNA dysregulation is implicated in a number of diseases, including cancer [46,47,48,49,50,51]. miRNAs are associated with cancers that are generally referred to as either oncomiRs (which are highly expressed often and can promote tumor development by the targeting of tumor suppressor genes) or tumor suppressive miRNA (which are often downregulated and inhibit cancer by regulating oncogenes [52]). Some cancer-associated miRNAs are known as context-dependent miRNAs. This is highly attributed to the fact that they can act in a tissue-specific manner so that single miRNAs can have either oncogenic or tumor suppressive roles in different cancers. Collectively, a surfeit of studies has reported alterations in miRNA expression in different types of cancers. Of particular interest, some miRNAs are related to cancer development, progression, and the response of the tumor to therapy [53,54,55]. Moreover, miRNAs can be secreted into body fluids and are referred to as circulating miRNAs [56]. They are highly stable and exist as free miRNA, or are released in exosomes [57,58]. The underlying mechanism of the relationship between tissue and circulating miRNA is not well known; yet, it seems that the extracellular miRNA levels reflect deregulated signaling pathways in cancer cells [59]. Finally, these small molecules, considered as one of the largest groups of gene regulators [60,61], are easily accessible, sensitive, specific, and stable; furthermore, they accordingly have a great potential to be considered as diagnostic, prognostic, and predictive biomarkers [46,49,62,63,64].

## 8. miRNA Implications in Breast Cancer

miRNA deregulation in BC was first reported in 2005 by Iorio, after which substantial evidence in research has depicted deregulated miRNA expression to be involved in BC initiation, progression, and metastasis [65,66,67,68,69,70]. Blenkiron et al., in 2007, analyzed the miRNA expression in human BCs and demonstrated distinct miRNA signatures for the different molecular BC subtypes [71,72]. The association of miRNA activity with BC biology and its behavior was further supported by the proof that miRNAs are implicated in the regulation of ER and HER2 [73]. Moreover, there is good evidence that miRNA expression differs between primary and metastatic BCs [74,75]. This consequently led researchers to consider miRNA signatures as potential biomarkers that would help to further the understanding of BC subtypes, as well as help to predict metastasis or therapeutic resistance, thus leading to prolonged patient survival [74,76,77].

The poor prognosis of TNBCs, as well as their aggressive behavior, frequent recurrence, and poor survival has provoked a great deal of studies, which investigated miRNA signatures as a tool through which to identify patients with TNBC apart from other BC subtypes, or from healthy individuals [60,78,79]. The dysregulation of certain miRNAs appears to also have a prognostic value in TNBCs [80]. Over the past few years, and with the advancement in sequencing, several studies identified miRNA changes that were associated with TNBC development and progression (detailed in Table 1).

Indeed, both tissue and circulating miRNAs are deregulated in TNBCs and are implicated with the various pathophysiological processes of initiation, development, and the progression of tumors, which may have the potential to help in the discovery of new diagnostic, prognostic, and therapeutic strategies.

**Figure 2:**
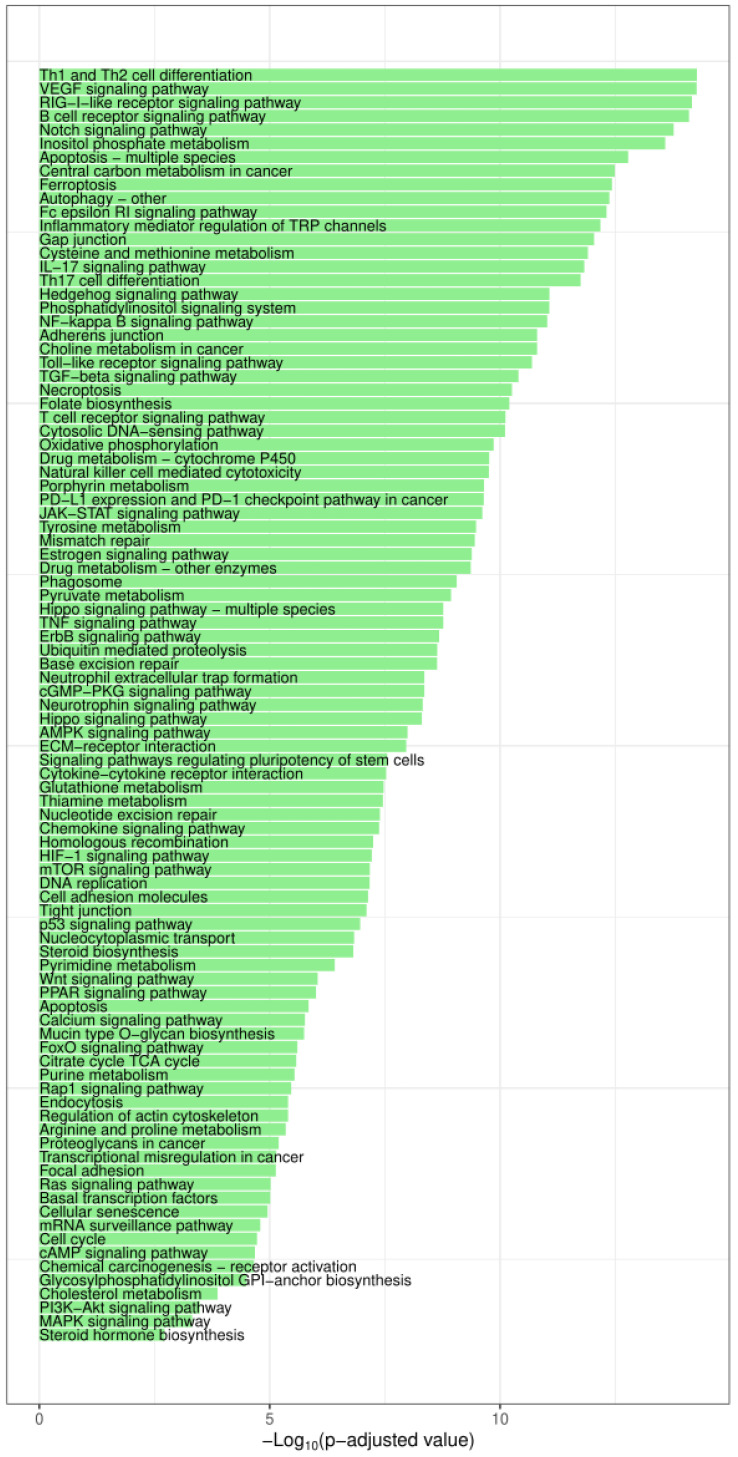
Bar plot depicting the significantly dysregulated pathways for all the dysregulated miRNAs in TNBCs, and adjusted for the decreasing *p*-values.

**Figure 3:**
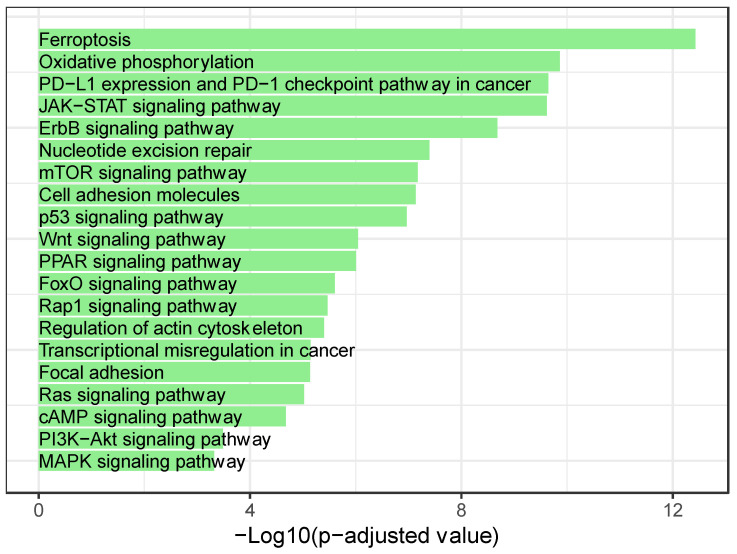
Bar plot depicting the significantly dysregulated pathways common to more than 20 miRNAs, and adjusted for the decreasing *p*-values.

In an effort to better understand how these miRNAs are having such an impact on TNBC carcinogenesis, we executed in-silico analysis to determine which pathways these miRNAs are regulating. First of all, we had to identify the predominant miRNAs in cases where they were not reported in the literature as 3p or 5p. This was conducted through the MiRBase Converter, which is embedded in the online miRNA Enrichment and Annotation Analaysis (miEAA) tools. We also checked the miRNA annotations through using the miRbase. After which, an over-representation analysis was performed for the dysregulated miRNAs by using (miEAA), as well as by selecting the Kyoto Encyclopedia of Genes and Genomes (KEGG) pathways database as a reference. Then, we manually filtered the results to include pathways that are solely associated to BC initiation, progression, and response to therapy. Also, only the significantly deregulated pathways were accounted for, whereby significance was determined based on there being a minimum of two miRNAs present in a pathway and those which had an adjusted *p*-value < 0.05 (Figure 2). Afterward, we identified the pathways that were found to be deregulated by a common set of more than 20 miRNAs (Figure 3). Out of the fifty-eight identified miRNA, twenty-one miRNA (hsa-miR-34a-5p; hsa-miR-93-5p; hsa-miR-124-3p; hsa-miR-15a-5p; hsa-miR-15b-5p; hsa-miR-16-5p; hsa-miR-195-5p; hsa-miR-145-5p; hsa-let-7e-5p; hsa-let-7b-5p; hsa-miR-301b-3p; hsa-miR-301a-3p; hsa-miR-30a-5p; hsa-miR-30c-5p; hsa-miR-9-5p; hsa-miR-210-3p; hsa-miR-19a-3p; hsa-miR-24-3p; hsa-miR-92a-3p; hsa-miR-222-3p; and hsa-miR-155-5p) were implicated in all of the pathways that are presented in Figure 3.

Our analysis reflects the complexity of miRNA interactions in TNBC carcinogenesis, i.e., where the existence of a set of signaling pathways that are reported to be implicated in TNBC hostility is indicated. Indeed, Javier Martinez et al. described epigenetic modifications as pivotal in TNBC development, as they appear to impact both oncogenes and tumor suppressor factors, which influence various molecular pathways such as WNT/β-catenin, MAPK, and PI3K-mTOR [237]. Another implication of WNT/β-catenin alongside JAK/STAT is that they regulate BC stem cell survival and thus raise the risk of TNBC relapse [238]. TNBCs’ genomic instability, metabolic plasticity, and mutation in genes (including p53 and MAPK influence signaling pathways) are associated with the immune response [239]. Also, several studies have described deregulated lipid metabolism as a contributor in cancer cell survival, and these studies also further showed that it was mediated by PPAR-α signaling pathway [240]. A major glitch in the treatment of TNBCs is reportedly chemoresistance. It is suggested that the EGFR-K-RAS-SIAH pathway activation is a major tumor driver in chemoresistant TNBC patients [241]; another pathway that is being investigated is cAMP and its anti-proliferative role [242]. Also, oxidative phosphorylation (OXPHOS) is associated with several cancers; however, TNBC patients with a higher expression of OXPHOS have been reported to have the worst outcome [243]. In addition, checkpoint inhibitor therapy holds promise, especially in the context of metastatic TNBCs where programmed death ligand 1 (PD-L1) and PD-1 pathways are being targeted by inhibitors in combination with other adopted treatments to try to alleviate patient response [244]. Finally, it is interesting to note that the ferroptosis pathway is largely represented. This type of cell death is increasingly studied in the context of cancer [245] in line with non-coding RNAs [246], as well as recently—in particular—in the ER−/AR+ BC subtype [247].

The predicted pathways in Figure 3 are not novel in terms of TNBC; yet, those pathways have also not been studied in terms of miRNA interaction. This sheds light on the importance of investigating the panels of miRNAs in the context of studying carcinogenesis pathways.

## 9. miRNA-Implications in AR+ Tumors

Recent investigations highlighted that AR expression may be regulated by a variety of miRNAs either directly or indirectly by affecting the expression of co-activators or co-repressors. The latter would shape the AR functions [248,249,250,251]. AR is a nuclear receptor made up of a single gene that is located on the X-chromosome [252,253,254]. Androgens are usually depicted as male hormones, yet they were found to also play important biological roles in female development and physiology [255]. Dehydroepiandrosterone sulphate (DHEAS), dehydroepiandrosterone (DHEA), androstenedione (A4), testosterone, and dihydrotestosterone (DHT) are kinds of androgenic hormones that are present in the blood stream [256].

First of all, a correlation between AR expression and miRNA is particularly depicted in prostate cancer (PC) [257,258]. This interaction was found to be associated with tumor initiation and development in PC. The androgen regulation of miRNAs was examined by Waltering et al. in 2011, where DHT was found to positively regulate 17 miRNAs, out of which only 4 (miR-10a, miR-141, miR-150, and miR-1225-5p) exhibited similar androgen regulation in both in vitro and in vivo studies [259]. AR activation in PC patients reduces miR-190a expression, thus enhancing tumor-free survival [250].

By contrast, the impact of AR in BC tumorigenesis remains controversial, for it was reported that women with increased levels of androgens have increased risk of BC, while it was also reported that AR expression is a favorable BC prognostic indicator (but it has to be noticed that this is mainly true in ER+ contexts [260,261,262]). The imbalance of miRNA levels in AR+ BC cells compared to AR− BC cells implies that miRNA has a crucial role in the function of AR in BCs [263]. However, studies on the miRNA–AR interactions in BCs are limited [257,258]. Some data indicate that miR-21, an oncomiR, is upregulated in hormone-dependent neoplasms including PC and BCs [264,265], and this is reported to reduce BC cell proliferation [130]. Interestingly, AR was found to repress the transcription of miR-21 expression [266]. This suggests that more has to be evaluated in this context.

Nevertheless, some studies have focused on BCs, especially ER− ones. Shi et al. performed miRNA expression profiling in ER−/AR+ BC and revealed a total of 153 differentially expressed miRNAs in AR+ compared to AR− BC. The most significantly upregulated miRNAs were miR-933 and miR-5793, and the most downregulated was miR-4792 [263]. miR-221 and 222 that are upregulated in BC and PC are considered as oncogenes where they promote proliferation. Of interest are the miRs that are repressed by AR [130]. Another miRNA that plays an essential role in ER−/AR+ cells is miR-30b, which has been reported to inhibit cell growth [267]. miR-9-5p has an inverse relationship with AR in BCs where it exerts an anti-proliferative role [268]. miR-328-3p suppression by DHT in MDA-MB-231, suppressed CD44 expression and consequently cell adhesion. Conversely, an opposite effect was obtained upon transfection with an AR antagonist, whereby the idea that miRNAs regulate BCs was emphasized [269]. miR-190a was previously reported to be implicated in BC metastasis [270]. miR-135b, a direct regulator of AR in PC cells, was shown to have a lower expression in ER+ breast tumors when compared to ER−, as well as a higher expression in AR-low BC patient samples. It also reduces proliferation in AR+ PC cells [260]. A study conducted by Guo et al. depicted that miR-520g-3p and miR-520h are both downregulated, and that they have a significant potential in AR+ TNBC diagnosis and prognosis [271]. miR-3163 that is downregulated in AR+ ER− tumors was found to have good prognostic value [272].

MABC/LARs, i.e., the scope of this review, are characterized by AR overexpression and hyperactivation. Little is known about the miRNAs associated with this subtype. This subtype has been investigated, in vitro, via BC cell lines, in which AR expression was shown to promote their growth [273]. Of interest, in the MDA-MB-453 cell line, is an MABC model, whereby the miRNA expression that was investigated by Lyu et al. in 2014 was found to reveal four upregulated miRNAs (let-7a, let-7b, let7-c, and let7-d), where let-7a decreased cell proliferation, invasion, and migration, as well as self-renewal capacities when treating cells with DHT. In addition, this process showed a better outcome in patients with invasive BCs [274,275]. AR activity is repressed indirectly by miR-let-7c [276]. Another study investigated the role of miR-30a in MDA-MB-453, after DHT treatment, and revealed that the stimulation of AR expression inhibits miR-30a and consequently suppresses cell growth [277]. In response to AR agonists, the miR-100 and miR-125 expression was significantly reduced in MDA-MB-453 BC cells, consequently leading to the increased expression of miR-100 and miR-125 target metalloprotease-13 (MMP13) [278].

A summary of the miRNAs implicated in AR+ BC and PC is summarized in Table 2.

## 10. Challenges

Despite the fact that BC is a highly investigated research topic, and that miRNAs can serve as a biomarker for BCs, the reports on MABC are not frequent, and—in most cases—not clear. MABC is often described as under TN in the literature but also as an ER− subtype with AR overexpression, yet the mention of the name itself is not stated. This also has an impact on the search for miRNA-MABC reports. Another obstacle with most of the miRNAs reported in the literature is the lack of full miRNA annotation. This requires the use of in silico programs to predict the isoforms of miRNAs, and these might not always end up in providing the isoform investigated in the literature. Moreover, miRNAs’ specificity is often questioned, since in many cases the data are unreproducible in different datasets. This could be explained by ethnic differences, age groups, or the standardization of miRNA quantification assays in all studies. In addition to this, pathway analysis is mostly dependent on algorithms and predictions. It is worthwhile to note that all the predicted actors need to be experimentally validated before clinical utility; however, this kind of analysis could be highly valuable for new hypotheses, and could promote further pathway explorations that could help with deciphering these poorly understood BCs. Furthermore, this inventory could be a starting point through which to develop new approaches for MABC/LAR BC subtypes by including the miRNA network in the picture.

## 11. Conclusions

Differential gene expression, epigenetic modification, IHC along with other current techniques in BC classification have revealed the huge heterogeneity of this disease. Therefore, understanding the different subtypes of BCs may benefit its diagnosis, prognosis, and therapeutics. This is essential in understanding poorly diagnosed and misclassified subtypes such as MABC/LARs, as well as the consequent impact on the health management of its corresponding patients. miRNAs are reported to be deregulated in various cancers, specifically in BC and in different BC subtypes (including ER−/AR+ ones). Hence, miRNAs are a highly stable and easily detectable molecule, and they may assist in a better understanding of MABC carcinogenesis. Thus, the verification of miRNA panels in MABC patients might create a distinctive definition of this subtype, and could depict an improved understanding of the signal networks driving the biology of MABCs. In addition to this, there is piling evidence of miRNA–AR interactions in development, as well as the progression of cancer that might elucidate on MABC initiation and progression. Moreover, specific miRNAs might actually serve as diagnostic or prognostic biomarkers, but more research needs to be conducted to verify the potential clinical application of these findings. Therefore, the search for ideal biomarkers necessitates the standardization of panels in different groups, and this is subject to continuous updates that are based on advances in research and molecular technology. In this context, exploring the state-of-the-art developments of miRNAs in the MABC/LAR subtype, and attempting to extract the main miRNAs of interest could shed light on this other level of complexity, as well as help to generate new hypotheses from new angles for approaching this BC subtype that is still poorly understood.

## Figures and Tables

**Table 1:** The dysregulated tissue and circulating miRNAs along with their various reported roles in TNBC carcinogenesis and their response to treatment.

miRNA Status	miRNA Annotation	Type	Role	Implications	Reference
Upregulated	miR-10b	Non-circulating	oncomiR	-Promotes proliferation, invasion, metastasis, and angiogenesis	[49,68,81,82]
	miR-181	Non-circulating	oncomiR	-Repressed by ER -Regulates the genes involved in cell growth and proliferation, including the progesterone receptor gene (a key player in estrogen signaling)	[68,83,84]
	miR-301	Non-circulating	oncomiR	-Correlates with a poor prognosis of TNBCs -Promotes the development of BCs	[85,86,87]
	miR-629-3p	Non-circulating	oncomiR	-Serves as a biomarker and a therapeutic target for lung metastasis in TNBCs	[88]
	miR-454	Non-circulating	oncomiR	-Associated with a poor prognosis and overall survival in TNBPC patients	[89]
	miR-301a	Non-circulating	oncomiR	-Correlated with a decreased overall survival and poor prognosis in TNBCs	[85,90]
	miR-182-5p	Non-circulating	oncomiR	-Promotes the proliferation and invasion of TNBCs -Associated with DNA damage repair -Correlated with cell proliferation and apoptosis	[91,92]
	miR-96-5p	Non-circulating	oncomiR	-Plays an important role in proliferation	[93]
	miR-135b	Non-circulating	oncomiR/ Suppressor	-Controls proliferation and invasion -Contributes to tumor development and progression -Worse survival in ER patients	[94,95,96]
	miR-138	Non-circulating	oncomiR	-Poor prognosis -Supports cell survival in cultures	[97]
	miR-20a-5p	Non- circulating	oncomiR	-Enhances metastasis -Implicated in apoptosis	[98,99]
	miR-455-3p	Non- circulating	oncomiR	-Improves metastasis -Increases proliferation	[100]
	miR146b-5p	Non-circulating	oncomiR	-Increases proliferation	[101]
	miR-324-5p	Non-circulating	oncomiR	-Implicated in apoptosis	[98]
	miR-939	Non-circulating	oncomiR	-Contributes to metastatic processes	[102]
	miR-362-5p	Non-circulating	oncomiR	-Facilitates proliferation and chemoresistance -Short overall survival	[103,104]
	miR-493	Non-circulating	Suppressor	-Better survival -Suppresses the invasiveness and tumorigenicity of BC cells	[105,106]
	miR-638	Non-circulating	Suppressor	-Better survival	[107]
	miR-146a	Non-circulating	Suppressor	-Better survival	[107]
	miR-182-3p	Non-circulating	Suppressor	-Reduces cell growth and activates apoptosis -Induces tumor inhibition in TNBCs	[108]
	miR-30	Non-circulating	Suppressor	-Activates p53 -Associated with good prognosis -miR-30c serves as an independent predictor in the clinical therapy of ER+ BC -Reduces cell proliferation and invasion in TNBCs	[68,109,110,111,112]
	miR-518a-3p	Non-circulating	Suppressor	-Inhibits cell migration and invasion -Better overall survival	[113]
	miR-522	Non-circulating	oncomiR	-Implicated in proliferation, invasion, and migration -High incidence of lymph node metastasis -Poor overall survival	[114]
	miR-934	Non-circulating	oncomiR	Cell proliferation	[115,116]
	miR-93-5p	Circulating	oncomiR	-Promotes chemoresistance -Acts as a diagnostic biomarker in TNBCs -Involved in TNBC metastasis and progression -Poor overall survival	[117,118,119]
	miR-105	Circulating	oncomiR	-Promotes metastasis, stemness, and chemoresistance -Poor overall survival	[118,120]
	miR-19a	Circulating	oncomiR	-Regulates anti-tumor immunity -Poor overall survival	[117,121]
	miR-19b	Circulating	oncomiR	-Promotes cell proliferation -Poor overall survival	[117,122]
	miR-22	Circulating	oncomiR	-Involved in cancer drug resistance -Promotes EMT	[117,123,124,125]
	miR-25-3p	Circulating and non-circulating	oncomiR	-Implicated in the inhibition of apoptosis -Promotes TNBC cell proliferation	[117,126]
	miR-210	Circulating and non-circulating	oncomiR	-Involved in microtubule regulation, drug efflux metabolism, and the oxidative stress response -Involved in cell proliferation, migration, and invasion -Associated with poor clinical outcomes in ER+ BC -Modulates the immune response	[68,117,127,128,129]
	miR-21	Circulating and non-circulating	oncomiR	-Promotes metastasis and proliferation -A marker of aggressiveness -Potentially prognostic in TNBC tumor stromata	[68,109,130,131,132,133,134,135,136,137,138]
	miR-19	Circulating and non-circulating	oncomiR	-Promotes EMT, migration, and invasion -Potential candidate for the diagnosis of BC when using blood samples	[139,140]
	miR-182	Circulating and non-circulating	oncomiR	-Targets the FOXO3 transcription factor expression -Promotes the macrophage activation that initiates cancer development	[141,142]
	miR-24	Circulating and non-circulating	oncomiR	-Predictor of BC relapse -Induces chemotherapy resistance -Regulates the proliferation and invasion of BC	[68,84,143,144,145]
	miR-503-3p	Circulating and non-circulating	oncomiR	-Promotes EMT	[146]
	miR-92	Circulating and non-circulating	oncomiR	-Enhances proliferation and migration	[147,148]
	miR-221/222	Circulating and non-circulating	oncomiR/ Suppressor	-Promotes EMT -Restores the expression of ER	[68,149,150,151,152]
	miR-155	Circulating and non-circulating	oncomiR/ Suppressor	-Cancer progression -Inversely correlated with the EMT in TNBCs -Associated with better clinical outcome in TNBCs -Enhances the antitumor immune response -Reverses paclitaxel resistance -A predictor of BC relapse	[53,68,109,153,154,155]
	miR-27b-3p	Circulating and non-circulating	oncomiR/ Suppressor	-A predictor of poor prognosis in invasive ductal TNBCs -Promotes tumor progression by inhibiting the peroxisome proliferator-activated receptor gamma in TNBCs	[156,157]
	miR-29a	Circulating and non-circulating	oncomiR/ Suppressor	-Promotes EMT, migration, and invasion by downregulating histone H4K20 trimethylation in TNBCs and ER+ cell lines -Decreases invasive BC cell proliferation, migration, and invasion in invasive breast cancers	[68,136,158,159]
	miR-200 family	Circulating and non-circulating	oncomiR/ Suppressor	-Promotes metastasis -Promotes EMT in aggressive cancers -Inhibits the growth and metastasis of claudin-low mammary cancers (TNBCs)	[160,161,162,163]
	miR-107	Circulating and non-circulating	oncomiR/ Suppressor	-Inhibits proliferation and migration -Associated with cell cycles, migration, invasion, revascularization, prognosis, and chemosensitivity -Improves overall survival	[98,164,165,166]
	miR-9	Circulating and non-circulating	oncomiR/Suppressor	-Associated with poor disease-free survival and distant-free survival -Enhances cell motility invasion and angiogenesis -Inhibits cell proliferation	[49,68,155,167,168]
Downregulated	miR-29c	Non-circulating	Suppressor	-Correlated with poor overall survival -Its loss is associated with the early development of TNBCs	[169]
	miR-17-5p	Non-circulating	Suppressor	-Prognostic factor for TNBCs	[170]
	miR-148a	Non-circulating	Suppressor	-Suppresses metastasis in vitro by reducing extravasation -Poor prognosis in basal and luminal B subtypes	[171]
	miR-126-5p	Non-circulating	Suppressor	-Impedes the metastasis of non-small cell lungs	[172]
	miR-1976	Non-circulating	Suppressor	-Bad overall survival -Promotes EMT	[173]
	miR-190a	Non-circulating	Suppressor	-Suppresses metastasis and angiogenesis -Correlated with a better overall survival	[96,174,175]
	miR-139-5p	Non-circulating	oncomiR	-Implicated in metastasis and chemoresistance	[176]
	miR-136-5p	Non-circulating	oncomiR	-Suppresses tumor invasion and metastasis	[96,177]
	miR-770-5p	Non-circulating	oncomiR	-Implicated in chemoresistance	[178]
	miR-4306	Non-circulating	oncomiR	-Lymph node metastasis -Poor survival -Promotes TNBC cell proliferation -Invasion and migration	[179]
	miR-196a-3p	Non-circulating	oncomiR	-Associated with lymph node metastasis -Pathological differentiation	[180]
	miR486-5p	Non-circulating	oncomiR	-Implicated in metastasis and chemoresistance	[181,182,183]
	miR-185	Non-circulating	Suppressor	-Inhibits TNBC cell proliferation	[184]
	miR-34	Non-circulating	Suppressor	-Induces apoptosis, cell cycle arrest, or senescence -Regulates cell growth, migration, invasion, angiogenesis, as well as epigenetic silencing and methylation -Promotes EMT	[49,68,109,185,186,187,188]
	miR-127	Non-circulating	Suppressor	-Suppresses proliferation, migration, and invasion -Sensitizes TNBC cells to chemotherapy	[189]
	miR-93	Non-circulating	Suppressor	-Suppresses tumor development -Enhances chemosensitivity -Mediates immunoregulation in BCs	[68,190,191,192]
	miR-124	Non-circulating	Suppressor	-Suppresses bone metastasis by repressing Interleukin-11	[193]
	miR-126	Non-circulating	Suppressor	-Associated with decreased cell proliferation -Targets the VEGF in MCF-7 cells -Inhibits the migration, invasion, and angiogenesis of TNBCs	[68,194,195,196,197]
	miR-133	Non-circulating	Suppressor	-Inhibits the growth of TNBCs	[198]
	miR-15/16	Non-circulating	Suppressor	-Inhibits cell proliferation in TNBCs -Controls angiogenesis	[199,200]
	miR-329	Non-circulating	Suppressor	-Correlates with metastasis	[201]
	miR-29a	Non-circulating	Suppressor	-Serves as a biomarker for BC diagnosis	[202]
	miR-4458	Non-circulating	Suppressor	-Regulates proliferation and apoptosis	[203]
	miR-4417	Non-circulating	Suppressor	-Prognostic biomarker for TNBCs	[204]
	miR-206	Non-circulating	oncomiR/Suppressor	-Promotes cancer progression in TNBCs and HER2+ BC by targeting neurokinin-1 receptor -Inhibits stemness and metastasis by targeting the MKL1/IL11 pathway -Suppresses EMT by targeting the TGF-β pathway in ER+ BC	[68,109,205,206,207]
	miR-31	Non-circulating	oncomiR/ Suppressor	-Correlated with poor prognosis	[208]
	miR-2117	Non- circulating	oncomiR	-Poor survival -Large tumor size	[116]
	miR-519c-3p	Non-circulating	oncomiR	-Associated with a large tumor size	[116]
	miR-873-5p	Non-circulating	Suppressor	-Promotes tumor development and metastasis	[209]
	miR-133	Non-circulating	oncomiR	-Induces proliferation and colony formation	[198]
	miR-585	Non-circulating	oncomiR	-Promotes cell proliferation, migration, and invasion -Significantly associated with poor prognosis	[210]
	miR-367	Circulating	Suppressor	-Regulates metastasis	[211]
	miR-494-3p	Circulating	oncomiR	-Implicated in immune system response	[212]
	miR-342	Circulating	Suppressor	-Biomarker for TNBCs	[168]
	miR-205	Circulating	oncomiR/ Suppressor	-Targets AR -A predictive marker of lymph node metastasis in luminal B- HER2+BC subtypes -miR-205-5p inhibits the proliferation and chemoresistance in TNBCs by targeting the HOXD9-Snail-1 axis -Expression decreases from less aggressive to more aggressive TNBCs -Inhibits proliferation and induces the EMT in TNBCs	[213,214,215,216]
	miR-199a	Circulating	oncomiR	-Affects chemosensitivity	[117,120]
	miR-195	Circulating and non-circulating	Suppressor	-Inhibits cell proliferation, glycolysis, and overall survival in ER+ BC -Differentiates metastatic BCs from the local luminal	[217,218]
	miR-205	Non-circulating	oncomiR	-Inversely associated with the tumor stage and distal metastasis of TNBCs -Poor prognosis	[219]
	Let-7 family	Circulating and non-circulating	Suppressor	-Suppresses invasion and migration -Regulates cancer stem cell properties (self-renewal, de-differentiation, and therapy resistance)	[117,220,221,222]
	miR-145	Circulating and non-circulating	Suppressor	-Suppresses metastasis and angiogenesis -Inhibits BC progression by inhibiting SOX2 -Diagnostic biomarker -Inhibits apoptosis by targeting cIAP1 (the cellular inhibitor of apoptosis)	[223,224,225,226]
	miR-335	Circulating and non-circulating	Suppressor	-Suppresses the immune escape in TNBCs -Enhances sensitivity to treatment and chemotherapy	[202,227,228,229]
	miR-128	Circulating and non-circulating	Suppressor	-Suppresses metastasis by targeting metadherin -Regulates glucose metabolism and proliferation in TNBCs	[230,231]
	miR-365	Circulating and non-circulating	Suppressor	-Anti-proliferative role -Controls invasion	[95,232]
	miR-503	Circulating and non-circulating	oncomiR/ Suppressor	-Enhances metastasis in metastatic BCs by activating the TGF-β pathway -Suppresses metastasis in ER+ BC cells -Inhibits proliferation by suppressing the CCND1 expression in BCs -Loss of miR-503 leads to chemoresistance	[233,234,235,236]

**Table 2:** Dysregulated tissue and the circulating miRNAs along with their various reported roles in AR+ BC and PC carcinogenesis, as well as their response to treatment.

Cancer Type	miRNA Status	miRNA Annotation	Type	Role	Implications of miRNA–AR Interaction	References
Breast cancer	Upregulated	miR-100	Non-circulating	Suppressor	-Extracellular release of MMP-13	[278,279]
		miR-125	Non-circulating	Suppressor	-Extracellular release of MMP-13	[278]
		miR-205	Non-circulating	oncomiR	-Metastasis	[213]
		miR-204	Non-circulating	Suppressor	-Promotes EMT	[280]
		miR-363	Non-circulating	oncomiR/ Suppressor	-AR induces miR-363 expression	[281]
		miR-let-7a	Non-circulating	Suppressor	-Tumor suppression, and AR induces a negative correlation between the expression of miR-let-7a and its target oncogenes of CMYC and KRAS	[274,275]
		miR-328-3p	Non-circulating	oncomiR	-Partially mediates the AR regulation of BCs	[269]
	Downregulated	miR-30a	Non-circulating	Suppressor	-Positive feedback mechanism -Suppresses cell growth	[282]
		miR-3163	Non-circulating	Suppressor	-Good prognostic role	[272]
		miR-520g-3p and miR-520h	Non-circulating	oncomiR	-Prognostic and diagnostic markers	[278]
	Differentially expressed	153 differentially expressed miRNAs in AR+ vs. AR− BC cell lines (miR-143, -4792,-145, -31, -30c, -30b-3p, 199a, and -181 downregulated in AR+ cells, while miR-933 and -5793 upregulated)	Non-circulating	oncomiR/ Suppressor	-The AR-mediated regulation of BCs is promoted by miRNAs	[263]
Prostate cancer	Upregulated	miR-17-92a	Non-circulating	oncomiR	-AR upregulates the expression of the miR-17-92a cluster	[281]
		miR-221/222	Non-circulating	oncomiR	-AR represses these miRNAs	[236]
		miR-190a	Non-circulating	oncomiR	-Contributes to tumor growth -Prognostic biomarker	[270]
	Downregulated	miR-760	Non-circulating	Suppressor	-AR downregulates miR-760, thus promoting PC growth	[283]
		miR-1205	Non-circulating	Suppressor	-Tumor suppressor	[284]
	Differentially expressed	miR-25 and miR-92b (downregulated) miR-3195, miR-3687, and miR-4417 (upregulated)	Non-circulating	oncomiR/ Suppressor	-AR upregulates the expression of these miRNAs	[285]
		miR-210-3p, miR-23c, miR-592, and miR-93-5	Circulating and non-circulating	oncomiR/ Suppressor	-Diagnostic biomarker	[286]

## Data Availability

All new data generated by in silico analysis in this study is already reported in this review.

## References

[1] WHO Cancer. https://www.who.int/news-room/fact-sheets/detail/cancer.

[2] Giaquinto A.N., Sung H., Miller K.D., Kramer J.L., Newman L.A., Minihan A., Jemal A., Siegel R.L. (2022). Breast Cancer Statistics, 2022. CA A Cancer J. Clin..

[3] Pfeiffer R.M., Webb-Vargas Y., Wheeler W., Gail M.H. (2018). Proportion of US trends in breast cancer incidence attributable to long-term changes in risk factor distributions. Cancer Epidemiol. Biomark. Prev..

[4] Riggio A.I., Varley K.E., Welm A.L. (2021). The lingering mysteries of metastatic recurrence in breast cancer. Br. J. Cancer.

[5] Koboldt D.C., Fulton R.S., Mclellan M.D., Schmidt H., Kalicki-Veizer J., McMichael J.F., Fulton L.L., Dooling D.J., Ding L., Elaine R. (2012). Comprehensive molecular portraits of human breast tumours. Nature.

[6] Board E. (2019). Breast Tumours. WHO Classification of Tumours.

[7] Tsang J., Tse G.M. (2020). Molecular classification of breast cancer. Adv. Anat. Pathol..

[8] Chamalidou C., Fohlin H., Albertsson P., Arnesson L.G., Einbeigi Z., Holmberg E., Nordenskjöld A., Nordenskjöld B., Karlsson P., Linderholm B. (2021). Survival patterns of invasive lobular and invasive ductal breast cancer in a large population-based cohort with two decades of follow up. Breast.

[9] McCart Reed A.E., Kalinowski L., Simpson P.T., Lakhani S.R. (2021). Invasive lobular carcinoma of the breast: The increasing importance of this special subtype. Breast Cancer Res..

[10] Perou C.M., Sørlie T., Eisen M.B., Van De Rijn M., Jeffrey S.S., Rees C.A., Pollack J.R., Ross D.T., Johnsen H., Akslen L.A. (2000). Molecular portraits of human breast tumours. Nature.

[11] Tan P.H., Ellis I., Allison K., Brogi E., Fox S.B., Lakhani S., Lazar A.J., Morris E.A., Sahin A., Salgado R. (2020). The 2019 World Health Organization classification of tumours of the breast. Histopathology.

[12] Goldhirsch A., Winer E.P., Coates A., Gelber R., Piccart-Gebhart M., Thürlimann B., Senn H.-J., Albain K.S., André F., Bergh J. (2013). Personalizing the treatment of women with early breast cancer: Highlights of the St Gallen International Expert Consensus on the Primary Therapy of Early Breast Cancer 2013. Ann. Oncol..

[13] Orrantia-Borunda E., Anchondo-Nuñez P., Acuña-Aguilar L.E., Gómez-Valles F.O., Ramírez-Valdespino C.A. (2022). Subtypes of Breast Cancer. Breast Cancer.

[14] Inic Z., Zegarac M., Inic M., Markovic I., Kozomara Z., Djurisic I., Inic I., Pupic G., Jancic S. (2014). Difference between luminal A and luminal B subtypes according to Ki-67, tumor size, and progesterone receptor negativity providing prognostic information. Clin. Med. Insights Oncol..

[15] Figueroa-Magalhães M.C., Jelovac D., Connolly R.M., Wolff A.C. (2014). Treatment of HER2-positive breast cancer. Breast.

[16] Lakhani S.R., Ellis I.O., Schnitt S., Tan P.H., van de Vijver M. (2012). WHO Classification of Tumours of the Breast.

[17] Lehmann B.D., Bauer J.A., Chen X., Sanders M.E., Chakravarthy A.B., Shyr Y., Pietenpol J.A. (2011). Identification of human triple-negative breast cancer subtypes and preclinical models for selection of targeted therapies. J. Clin. Investig..

[18] Bareche Y., Buisseret L., Gruosso T., Girard E., Venet D., Dupont F., Desmedt C., Larsimont D., Park M., Rothé F. (2019). Unraveling Triple-Negative Breast Cancer Tumor Microenvironment Heterogeneity: Towards an Optimized Treatment Approach. JNCI J. Natl. Cancer Inst..

[19] Kim J., Yu D., Kwon Y., Lee K.S., Sim S.H., Kong S.-Y., Lee E.S., Park I.H., Park C. (2020). Genomic Characteristics of Triple-Negative Breast Cancer Nominate Molecular Subtypes That Predict Chemotherapy ResponseTNBC Subtypes and Chemotherapy Response. Mol. Cancer Res..

[20] Lehmann B.D., Jovanović B., Chen X., Estrada M.V., Johnson K.N., Shyr Y., Moses H.L., Sanders M.E., Pietenpol J.A. (2016). Refinement of triple-negative breast cancer molecular subtypes: Implications for neoadjuvant chemotherapy selection. PLoS ONE.

[21] Darb-Esfahani S., von Minckwitz G., Denkert C., Ataseven B., Högel B., Mehta K., Kaltenecker G., Rüdiger T., Pfitzner B., Kittel K. (2014). Gross cystic disease fluid protein 15 (GCDFP-15) expression in breast cancer subtypes. BMC Cancer.

[22] Mazoujian G., Pinkus G., Davis S., Haagensen D. (1983). Immunohistochemistry of a gross cystic disease fluid protein (GCDFP-15) of the breast. A marker of apocrine epithelium and breast carcinomas with apocrine features. Am. J. Pathol..

[23] Farmer P., Bonnefoi H., Becette V., Tubiana-Hulin M., Fumoleau P., Larsimont D., MacGrogan G., Bergh J., Cameron D., Goldstein D. (2005). Identification of molecular apocrine breast tumours by microarray analysis. Breast Cancer Res..

[24] Doane A.S., Danso M., Lal P., Donaton M., Zhang L., Hudis C., Gerald W.L. (2006). An estrogen receptor-negative breast cancer subset characterized by a hormonally regulated transcriptional program and response to androgen. Oncogene.

[25] Guedj M., Marisa L., De Reynies A., Orsetti B., Schiappa R., Bibeau F., Macgrogan G., Lerebours F., Finetti P., Longy M. (2012). A refined molecular taxonomy of breast cancer. Oncogene.

[26] Lehmann-Che J., Hamy A.-S., Porcher R., Barritault M., Bouhidel F., Habuellelah H., Leman-Detours S., De Roquancourt A., Cahen-Doidy L., Bourstyn E. (2013). Molecular apocrine breast cancers are aggressive estrogen receptor negative tumors overexpressing either HER2 or GCDFP15. Breast Cancer Res..

[27] D’Arcy C., Quinn C.M. (2019). Apocrine lesions of the breast: Part 2 of a two-part review. Invasive apocrine carcinoma, the molecular apocrine signature and utility of immunohistochemistry in the diagnosis of apocrine lesions of the breast. J. Clin. Pathol..

[28] Huang R., Han J., Liang X., Sun S., Jiang Y., Xia B., Niu M., Li D., Zhang J., Wang S. (2017). Androgen Receptor Expression and Bicalutamide Antagonize Androgen Receptor Inhibit β-Catenin Transcription Complex in Estrogen Receptor-Negative Breast Cancer. Cell Physiol. Biochem..

[29] Iacopetta D., Rechoum Y., Fuqua S.A. (2012). The role of androgen receptor in breast cancer. Drug Discov. Today Dis. Mech..

[30] Tsang J.Y., Ni Y.-B., Chan S.-K., Shao M.-M., Law B.K., Tan P.H., Tse G.M. (2014). Androgen receptor expression shows distinctive significance in ER positive and negative breast cancers. Ann. Surg. Oncol..

[31] Wardley A., Mueller V., Paplomata E., Crouzet L., Iqbal N., Aithal S., Block M., Cold S., By M.-A., Hahn O. (2021). Abstract PD13-04: Impact of tucatinib on health-related quality of life in patients with HER2+ metastatic breast cancer with stable and active brain metastases. Cancer Res..

[32] Bonnefoi H., Grellety T., Tredan O., Saghatchian M., Dalenc F., Mailliez A., L’haridon T., Cottu P., Abadie-Lacourtoisie S., You B. (2016). A phase II trial of abiraterone acetate plus prednisone in patients with triple-negative androgen receptor positive locally advanced or metastatic breast cancer (UCBG 12-1). Ann. Oncol..

[33] Grellety T., Callens C., Richard E., Briaux A., Vélasco V., Pulido M., Gonçalves A., Gestraud P., MacGrogan G., Bonnefoi H. (2019). Enhancing Abiraterone Acetate Efficacy in Androgen Receptor–positive Triple-negative Breast Cancer: Chk1 as a Potential TargetAbiraterone and Chk1 Inhibitor in AR-positive TNBC. Clin. Cancer Res..

[34] Wardley A., Cortes J., Provencher L., Miller K., Chien A.J., Rugo H.S., Steinberg J., Sugg J., Tudor I.C., Huizing M. (2021). The efficacy and safety of enzalutamide with trastuzumab in patients with HER2+ and androgen receptor-positive metastatic or locally advanced breast cancer. Breast Cancer Res. Treat..

[35] Gucalp A., Tolaney S., Isakoff S.J., Ingle J.N., Liu M.C., Carey L.A., Blackwell K., Rugo H., Nabell L., Forero A. (2013). Phase II trial of bicalutamide in patients with androgen receptor–positive, estrogen receptor–negative metastatic breast cancer. Clin. Cancer Res..

[36] Traina T.A., Miller K., Yardley D.A., Eakle J., Schwartzberg L.S., O’Shaughnessy J., Gradishar W., Schmid P., Winer E., Kelly C. (2018). Enzalutamide for the treatment of androgen receptor–expressing triple-negative breast cancer. J. Clin. Oncol..

[37] Burstein H., Curigliano G., Thürlimann B., Weber W., Poortmans P., Regan M., Senn H., Winer E., Gnant M., Aebi S. (2021). Customizing local and systemic therapies for women with early breast cancer: The St. Gallen International Consensus Guidelines for treatment of early breast cancer 2021. Ann. Oncol..

[38] Kono M., Fujii T., Lim B., Karuturi M.S., Tripathy D., Ueno N.T. (2017). Androgen receptor function and androgen receptor–targeted therapies in breast cancer: A review. JAMA Oncol..

[39] Felekkis K., Touvana E., Stefanou C., Deltas C. (2010). microRNAs: A newly described class of encoded molecules that play a role in health and disease. Hippokratia.

[40] Ha M., Kim V.N. (2014). Regulation of microRNA biogenesis. Nat. Rev. Mol. Cell Biol..

[41] Place R.F., Li L.-C., Pookot D., Noonan E.J., Dahiya R. (2008). MicroRNA-373 induces expression of genes with complementary promoter sequences. Proc. Natl. Acad. Sci. USA.

[42] Doench J.G., Sharp P.A. (2004). Specificity of microRNA target selection in translational repression. Genes. Dev..

[43] O’Day E., Lal A. (2010). MicroRNAs and their target gene networks in breast cancer. Breast Cancer Res..

[44] Vasudevan S., Tong Y., Steitz J.A. (2007). Switching from repression to activation: microRNAs can up-regulate translation. Science.

[45] Kurisetty V.V., Lakshmanaswamy R., Damodaran C. (2014). Pathogenic and therapeutic role of miRNAs in breast cancer. Front. Biosci..

[46] Bartel D.P. (2004). MicroRNAs: Genomics, biogenesis, mechanism, and function. Cell.

[47] Ardekani A.M., Naeini M.M. (2010). The role of microRNAs in human diseases. Avicenna J. Med. Biotechnol..

[48] Esteller M. (2011). Non-coding RNAs in human disease. Nat. Rev. Genet..

[49] Iorio M.V., Croce C.M. (2012). MicroRNA dysregulation in cancer: Diagnostics, monitoring and therapeutics. A comprehensive review. EMBO Mol. Med..

[50] Rupaimoole R., Calin G.A., Lopez-Berestein G., Sood A.K. (2016). miRNA deregulation in cancer cells and the tumor microenvironment. Cancer Discov..

[51] Bautista-Sánchez D., Arriaga-Canon C., Pedroza-Torres A., De La Rosa-Velázquez I.A., González-Barrios R., Contreras-Espinosa L., Montiel-Manríquez R., Castro-Hernández C., Fragoso-Ontiveros V., Álvarez-Gómez R.M. (2020). The promising role of miR-21 as a cancer biomarker and its importance in RNA-based therapeutics. Mol. Ther.-Nucleic Acids.

[52] Fridrichova I., Zmetakova I. (2019). MicroRNAs Contribute to Breast Cancer Invasiveness. Cells.

[53] Sun Y., Wang M., Lin G., Sun S., Li X., Qi J., Li J. (2012). Serum microRNA-155 as a potential biomarker to track disease in breast cancer. Chin. Sci..

[54] Wang H., Tan G., Dong L., Cheng L., Li K., Wang Z., Luo H. (2012). Circulating MiR-125b as a marker predicting chemoresistance in breast cancer. PLoS ONE.

[55] Fatica A., Bozzoni I. (2014). Long non-coding RNAs: New players in cell differentiation and development. Nat. Rev. Genet..

[56] Weber J.A., Baxter D.H., Zhang S., Huang D.Y., How Huang K., Jen Lee M., Galas D.J., Wang K. (2010). The microRNA spectrum in 12 body fluids. Clin. Chem..

[57] Théry C. (2011). Exosomes: Secreted vesicles and intercellular communications. F1000 Biol. Rep..

[58] Théry C., Zitvogel L., Amigorena S. (2002). Exosomes: Composition, biogenesis and function. Nat. Rev. Immunol..

[59] Wang J., Zhang K.-Y., Liu S.-M., Sen S. (2014). Tumor-associated circulating microRNAs as biomarkers of cancer. Molecules.

[60] Kahraman M., Röske A., Laufer T., Fehlmann T., Backes C., Kern F., Kohlhaas J., Schrörs H., Saiz A., Zabler C. (2018). MicroRNA in diagnosis and therapy monitoring of early-stage triple-negative breast cancer. Sci. Rep..

[61] Loh H.-Y., Norman B.P., Lai K.-S., Rahman N.M.A.N.A., Alitheen N.B.M., Osman M.A. (2019). The regulatory role of microRNAs in breast cancer. Int. J. Mol. Sci..

[62] Wiemer E.A. (2007). The role of microRNAs in cancer: No small matter. Eur. J. Cancer.

[63] He L., Hannon G.J. (2004). MicroRNAs: Small RNAs with a big role in gene regulation. Nat. Rev. Genet..

[64] Ho P.T.B., Clark I.M., Le L.T.T. (2022). MicroRNA-Based Diagnosis and Therapy. Int. J. Mol. Sci..

[65] Andorfer C.A., Necela B.M., Thompson E.A., Perez E.A. (2011). MicroRNA signatures: Clinical biomarkers for the diagnosis and treatment of breast cancer. Trends Mol. Med..

[66] Shi M., Guo N. (2009). MicroRNA expression and its implications for the diagnosis and therapeutic strategies of breast cancer. Cancer Treat. Rev..

[67] van Schooneveld E., Wildiers H., Vergote I., Vermeulen P.B., Dirix L.Y., Van Laere S.J. (2015). Dysregulation of microRNAs in breast cancer and their potential role as prognostic and predictive biomarkers in patient management. Breast Cancer Res..

[68] Bertoli G., Cava C., Castiglioni I. (2015). MicroRNAs: New Biomarkers for Diagnosis, Prognosis, Therapy Prediction and Therapeutic Tools for Breast Cancer. Theranostics.

[69] Kurozumi S., Yamaguchi Y., Kurosumi M., Ohira M., Matsumoto H., Horiguchi J. (2017). Recent trends in microRNA research into breast cancer with particular focus on the associations between microRNAs and intrinsic subtypes. J. Hum. Genet..

[70] Iorio M.V., Ferracin M., Liu C.-G., Veronese A., Spizzo R., Sabbioni S., Magri E., Pedriali M., Fabbri M., Campiglio M. (2005). MicroRNA gene expression deregulation in human breast cancer. Cancer Res..

[71] Blenkiron C., Goldstein L.D., Thorne N.P., Spiteri I., Chin S.F., Dunning M.J., Barbosa-Morais N.L., Teschendorff A.E., Green A.R., Ellis I.O. (2007). MicroRNA expression profiling of human breast cancer identifies new markers of tumor subtype. Genome Biol..

[72] Wang W., Luo Y.-P. (2015). MicroRNAs in breast cancer: Oncogene and tumor suppressors with clinical potential. J. Zhejiang Univ.-SCIENCE B.

[73] Castañeda C.A., Agullo-Ortuño M.T., Fresno Vara J.A., Cortes-Funes H., Gomez H.L., Ciruelos E. (2011). Implication of miRNA in the diagnosis and treatment of breast cancer. Expert Rev. Anticancer Ther..

[74] Baffa R., Fassan M., Volinia S., O’Hara B., Liu C.G., Palazzo J.P., Gardiman M., Rugge M., Gomella L.G., Croce C.M. (2009). MicroRNA expression profiling of human metastatic cancers identifies cancer gene targets. J. Pathol. A J. Pathol. Soc. Great Br. Irel..

[75] Rahman M.M., Brane A.C., Tollefsbol T.O. (2019). MicroRNAs and epigenetics strategies to reverse breast cancer. Cells.

[76] Shi M., Liu D., Duan H., Shen B., Guo N. (2010). Metastasis-related miRNAs, active players in breast cancer invasion, and metastasis. Cancer Metastasis Rev..

[77] Guttery D.S., Blighe K., Page K., Marchese S.D., Hills A., Coombes R.C., Stebbing J., Shaw J.A. (2013). Hide and seek: Tell-tale signs of breast cancer lurking in the blood. Cancer Metastasis Rev..

[78] de Brot S., Rutland C.S., Mongan N.P., James V., Chakrabarti D.J., Mitra D.S. (2018). Chapter 20—Epigenetic Control of MicroRNA Expression and Cancer. Cancer and Noncoding RNAs.

[79] Gonçalves H., Guerra M.R., Duarte Cintra J.R., Fayer V.A., Brum I.V., Bustamante Teixeira M.T. (2018). Survival study of triple-negative and non–triple-negative breast cancer in a Brazilian cohort. Clin. Med. Insights Oncol..

[80] Piasecka D., Braun M., Kordek R., Sadej R., Romanska H. (2018). MicroRNAs in regulation of triple-negative breast cancer progression. J. Cancer Res. Clin. Oncol..

[81] Wang D., Wang Z., Zhang L., Sun S. (2021). LncRNA PDCD4-AS1 alleviates triple negative breast cancer by increasing expression of IQGAP2 via miR-10b-5p. Transl. Oncol..

[82] Raval A., Joshi J., Shah F. (2022). Significance of metastamiR-10b in breast cancer therapeutics. J. Egypt. Natl. Cancer Inst..

[83] Maillot G., Lacroix-Triki M., Pierredon S., Gratadou L., Schmidt S., Bénès V., Roché H., Dalenc F., Auboeuf D., Millevoi S. (2009). Widespread Estrogen-Dependent Repression of microRNAs Involved in Breast Tumor Cell GrowthEstrogen-Regulated MicroRNAs. Cancer Res..

[84] Sochor M., Basova P., Pesta M., Dusilkova N., Bartos J., Burda P., Pospisil V., Stopka T. (2014). Oncogenic microRNAs: miR-155, miR-19a, miR-181b, and miR-24 enable monitoring of early breast cancer in serum. BMC Cancer.

[85] Yu H., Li H., Qian H., Jiao X., Zhu X., Jiang X., Dai G., Huang J. (2014). Upregulation of miR-301a correlates with poor prognosis in triple-negative breast cancer. Med. Oncol..

[86] Lu X., Duan J., Zhou R., Xu Y. (2021). MiR-301b-3p promotes the occurrence and development of breast cancer cells via targeting HOXA5. Crit. Rev.™ Eukaryot. Gene Expr..

[87] Zheng J.-Z., Huang Y.-N., Yao L., Liu Y.-R., Liu S., Hu X., Liu Z.-B., Shao Z.-M. (2018). Elevated miR-301a expression indicates a poor prognosis for breast cancer patients. Sci. Rep..

[88] Wang J., Song C., Tang H., Zhang C., Tang J., Li X., Chen B., Xie X. (2017). miR-629-3p may serve as a novel biomarker and potential therapeutic target for lung metastases of triple-negative breast cancer. Breast Cancer Res..

[89] Cao Z., Li J., Yao L. (2016). High expression of microRNA-454 is associated with poor prognosis in triple-negative breast cancer. Oncotarget.

[90] Ma F., Zhang J., Zhong L., Wang L., Liu Y., Wang Y., Peng L., Guo B. (2014). Upregulated microRNA-301a in breast cancer promotes tumor metastasis by targeting PTEN and activating Wnt/β-catenin signaling. Gene.

[91] Wu X., Chen H., Wu M., Peng S., Zhang L. (2020). Downregulation of miR-182-5p inhibits the proliferation and invasion of triple-negative breast cancer cells through regulating TLR4/NF-κB pathway activity by targeting FBXW7. Ann. Transl. Med..

[92] Wei Q., Lei R., Hu G. (2015). Roles of miR-182 in sensory organ development and cancer. Thorac. Cancer.

[93] Mendes D.C.C., Filho C.M.C.C., Garcia N., Ricci M.D., Soares J.M., Carvalho K.C., Baracat E.C. (2023). Could be FOXO3a, miR-96-5p and miR-182-5p useful for Brazilian women with luminal A and triple negative breast cancers prognosis and target therapy?. Clinics.

[94] Manic G., Obrist F., Sistigu A., Vitale I. (2015). Trial watch: Targeting ATM–CHK2 and ATR–CHK1 pathways for anticancer therapy. Mol. Cell. Oncol..

[95] Bertoli G., Cava C., Corsi F., Piccotti F., Martelli C., Ottobrini L., Vaira V., Castiglioni I. (2021). Triple negative aggressive phenotype controlled by miR-135b and miR-365: New theranostics candidates. Sci. Rep..

[96] Paszek S., Gabło N., Barnaś E., Szybka M., Morawiec J., Kołacińska A., Zawlik I. (2017). Dysregulation of microRNAs in triple-negative breast cancer. Ginekol. Pol..

[97] Nama S., Muhuri M., Di Pascale F., Quah S., Aswad L., Fullwood M., Sampath P. (2019). MicroRNA-138 is a Prognostic Biomarker for Triple-Negative Breast Cancer and Promotes Tumorigenesis via TUSC2 repression. Sci. Rep..

[98] Hong H.-C., Chuang C.-H., Huang W.-C., Weng S.-L., Chen C.-H., Chang K.-H., Liao K.-W., Huang H.-D. (2020). A panel of eight microRNAs is a good predictive parameter for triple-negative breast cancer relapse. Theranostics.

[99] Li X., Wu B., Chen L., Ju Y., Li C., Meng S. (2017). Urokinase-type plasminogen activator receptor inhibits apoptosis in triple-negative breast cancer through miR-17/20a suppression of death receptors 4 and 5. Oncotarget.

[100] Li Z., Meng Q., Pan A., Wu X., Cui J., Wang Y., Li L. (2017). MicroRNA-455-3p promotes invasion and migration in triple negative breast cancer by targeting tumor suppressor EI24. Oncotarget.

[101] Garcia A.I., Buisson M., Bertrand P., Rimokh R., Rouleau E., Lopez B.S., Lidereau R., Mikaélian I., Mazoyer S. (2011). Down-regulation of BRCA1 expression by miR-146a and miR-146b-5p in triple negative sporadic breast cancers. EMBO Mol. Med..

[102] Di Modica M., Regondi V., Sandri M., Iorio M.V., Zanetti A., Tagliabue E., Casalini P., Triulzi T. (2017). Breast cancer-secreted miR-939 downregulates VE-cadherin and destroys the barrier function of endothelial monolayers. Cancer Lett..

[103] Xia J.-T., Chen L.-Z., Jian W.-H., Wang K.-B., Yang Y.-Z., He W.-L., He Y.-L., Chen D., Li W. (2014). MicroRNA-362 induces cell proliferation and apoptosis resistance in gastric cancer by activation of NF-κB signaling. J. Transl. Med..

[104] Zhang X., He Q., Sun L., Zhang Y., Qin S., Fan J., Wang J. (2019). Comparing MicroRNA profilings of purified HER-2-negative and HER-2-positive cells validates miR-362-5p/Sema3A as characteristic molecular change in triple-negative breast cancers. Dis. Markers.

[105] Yao L., Liu Y., Cao Z., Li J., Huang Y., Hu X., Shao Z. (2018). MicroRNA-493 is a prognostic factor in triple-negative breast cancer. Cancer Sci..

[106] Zhao L., Feng X., Song X., Zhou H., Zhao Y., Cheng L., Jia L. (2016). miR-493-5p attenuates the invasiveness and tumorigenicity in human breast cancer by targeting FUT4. Oncol. Rep..

[107] Zavala V., Perez-Moreno E., Tapia T., Camus M., Carvallo P. (2016). miR-146a and miR-638 in BRCA1-deficient triple negative breast cancer tumors, as potential biomarkers for improved overall survival. Cancer Biomark..

[108] Dinami R., Pompili L., Petti E., Porru M., D’Angelo C., Di Vito S., Rizzo A., Campani V., De Rosa G., Bruna A. (2023). MiR-182-3p targets TRF2 and impairs tumor growth of triple-negative breast cancer. EMBO Mol. Med..

[109] Tang J., Ahmad A., Sarkar F.H. (2012). The role of microRNAs in breast cancer migration, invasion and metastasis. Int. J. Mol. Sci..

[110] Shukla K., Sharma A.K., Ward A., Will R., Hielscher T., Balwierz A., Breunig C., Münstermann E., König R., Keklikoglou I. (2015). MicroRNA-30c-2-3p negatively regulates NF-κB signaling and cell cycle progression through downregulation of TRADD and CCNE1 in breast cancer. Mol. Oncol..

[111] Di Gennaro A., Damiano V., Brisotto G., Armellin M., Perin T., Zucchetto A., Guardascione M., Spaink H.P., Doglioni C., Snaar-Jagalska B.E. (2018). A p53/miR-30a/ZEB2 axis controls triple negative breast cancer aggressiveness. Cell Death Differ..

[112] Rodríguez-González F.G., Sieuwerts A.M., Smid M., Look M.P., Meijer-van Gelder M.E., de Weerd V., Sleijfer S., Martens J.W., Foekens J.A. (2011). MicroRNA-30c expression level is an independent predictor of clinical benefit of endocrine therapy in advanced estrogen receptor positive breast cancer. Breast Cancer Res. Treat..

[113] Gan L., Yang H., Xiong Z., Yang Z., Wang T., Lyu G. (2020). miR-518a-3p Suppresses Triple-Negative Breast Cancer Invasion and Migration through Regulation of TMEM2. Technol. Cancer Res. Treat..

[114] Wang W., Zhang W., Wu J., Zhou Z., Ma J. (2022). miR-522 regulates cell proliferation, migration, invasion capacities and acts as a potential biomarker to predict prognosis in triple-negative breast cancer. Clin. Exp. Med..

[115] Castilla M.Á., López-García M.Á., Atienza M.R., Rosa-Rosa J.M., Díaz-Martín J., Pecero M.L., Vieites B., Romero-Pérez L., Benítez J., Calcabrini A. (2014). VGLL1 expression is associated with a triple-negative basal-like phenotype in breast cancer. Endocr.-Relat. Cancer.

[116] Turkistani S., Sugita B.M., Fadda P., Marchi R., Afsari A., Naab T., Apprey V., Copeland R.L., Campbell M.C., Cavalli L.R. (2021). A panel of miRNAs as prognostic markers for African-American patients with triple negative breast cancer. BMC Cancer.

[117] Qattan A., Al-Tweigeri T., Alkhayal W., Suleman K., Tulbah A., Amer S. (2021). Clinical identification of dysregulated circulating microRNAs and their implication in drug response in triple negative breast cancer (TNBC) by target gene network and meta-analysis. Genes.

[118] Li H.-Y., Liang J.-L., Kuo Y.-L., Lee H.-H., Calkins M.J., Chang H.-T., Lin F.-C., Chen Y.-C., Hsu T.-I., Hsiao M. (2017). miR-105/93-3p promotes chemoresistance and circulating miR-105/93-3p acts as a diagnostic biomarker for triple negative breast cancer. Breast Cancer Res..

[119] Grossman R.L., Heath A.P., Ferretti V., Varmus H.E., Lowy D.R., Kibbe W.A., Staudt L.M. (2016). Toward a shared vision for cancer genomic data. N. Engl. J. Med..

[120] Zang C., Zhao F., Hua L., Pu Y. (2018). The miR-199a-3p regulates the radioresistance of esophageal cancer cells via targeting the AK4 gene. Cancer Cell Int..

[121] Anfossi S., Giordano A., Gao H., Cohen E.N., Tin S., Wu Q., Garza R.J., Debeb B.G., Alvarez R.H., Valero V. (2014). High serum miR-19a levels are associated with inflammatory breast cancer and are predictive of favorable clinical outcome in patients with metastatic HER2+ inflammatory breast cancer. PLoS ONE.

[122] Wishart D.S., Knox C., Guo A.C., Shrivastava S., Hassanali M., Stothard P., Chang Z., Woolsey J. (2006). DrugBank: A comprehensive resource for in silico drug discovery and exploration. Nucleic Acids Res..

[123] Lee K.-L., Kuo Y.-C., Ho Y.-S., Huang Y.-H. (2019). Triple-negative breast cancer: Current understanding and future therapeutic breakthrough targeting cancer stemness. Cancers.

[124] De Angelis M.L., Francescangeli F., Zeuner A. (2019). Breast cancer stem cells as drivers of tumor chemoresistance, dormancy and relapse: New challenges and therapeutic opportunities. Cancers.

[125] Song S.J., Poliseno L., Song M.S., Ala U., Webster K., Ng C., Beringer G., Brikbak N.J., Yuan X., Cantley L.C. (2013). MicroRNA-antagonism regulates breast cancer stemness and metastasis via TET-family-dependent chromatin remodeling. Cell.

[126] Chen H., Pan H., Qian Y., Zhou W., Liu X. (2018). MiR-25-3p promotes the proliferation of triple negative breast cancer by targeting BTG2. Mol. Cancer.

[127] Pasculli B., Barbano R., Rendina M., Fontana A., Copetti M., Mazza T., Valori V.M., Morritti M., Maiello E., Graziano P. (2019). Hsa-miR-210-3p expression in breast cancer and its putative association with worse outcome in patients treated with Docetaxel. Sci. Rep..

[128] Rothe F., Ignatiadis M., Chaboteaux C., Haibe-Kains B., Kheddoumi N., Majjaj S., Badran B., Fayyad-Kazan H., Desmedt C., Harris A.L. (2011). Global microRNA expression profiling identifies MiR-210 associated with tumor proliferation, invasion and poor clinical outcome in breast cancer. PLoS ONE.

[129] Bar I., Merhi A., Abdel-Sater F., Ben Addi A., Sollennita S., Canon J.-L., Delrée P. (2017). The MicroRNA miR-210 Is Expressed by Cancer Cells but Also by the Tumor Microenvironment in Triple-Negative Breast Cancer. J. Histochem. Cytochem..

[130] Casaburi I., Cesario G.M., Donà A., Rizza P., Aquila S., Avena P., Lanzino M., Pellegrino M., Vivacqua A., Tucci P. (2016). Androgens downregulate miR-21 expression in breast cancer cells underlining the protective role of androgen receptor. Oncotarget.

[131] Isaacs C., Hayes D.F., Stearns V. (2001). Prognostic Factors in Breast Cancer: Current and New Predictors of Metastasis. J. Mammary Gland. Biol. Neoplasia.

[132] Corcoran C., Friel A.M., Duffy M.J., Crown J., O’Driscoll L. (2011). Intracellular and extracellular microRNAs in breast cancer. Clin. Chem..

[133] Özgün A., Karagoz B., Bilgi O., Tuncel T., Baloglu H., Kandemir E.G. (2013). MicroRNA-21 as an indicator of aggressive phenotype in breast cancer. Oncol. Res. Treat..

[134] Dong G., Liang X., Wang D., Gao H., Wang L., Wang L., Liu J., Du Z. (2014). High expression of miR-21 in triple-negative breast cancers was correlated with a poor prognosis and promoted tumor cell in vitro proliferation. Med. Oncol..

[135] Pfeffer S.R., Yang C.H., Pfeffer L.M. (2015). The role of miR-21 in cancer. Drug Dev. Res..

[136] Wu Q., Lu Z., Li H., Lu J., Guo L., Ge Q. (2011). Next-generation sequencing of microRNAs for breast cancer detection. J. Biomed. Biotechnol..

[137] Volinia S., Calin G.A., Liu C.-G., Ambs S., Cimmino A., Petrocca F., Visone R., Iorio M., Roldo C., Ferracin M. (2006). A microRNA expression signature of human solid tumors defines cancer gene targets. Proc. Natl. Acad. Sci. USA.

[138] MacKenzie T.A., Schwartz G.N., Calderone H.M., Graveel C.R., Winn M.E., Hostetter G., Wells W.A., Sempere L.F. (2014). Stromal Expression of miR-21 Identifies High-Risk Group in Triple-Negative Breast Cancer. Am. J. Pathol..

[139] Liu A., Yang F., Huang L., Zhang L., Zhang J., Zheng R. (2019). Long non-coding RNA Tubulin Alpha 4B (TUBA4B) inhibited breast cancer proliferation and invasion by directly targeting miR-19. Eur. Rev. Med. Pharmacol. Sci..

[140] Zhao Q., Shen L., Lü J., Xie H., Li D., Shang Y., Huang L., Meng L., An X., Zhou J. (2022). A circulating miR-19b-based model in diagnosis of human breast cancer. Front. Mol. Biosci..

[141] Kandil N.S., Kandil L.S., Mohamed R., Selima M., El Nemr M., Barakat A.R., Alwany Y.N. (2022). The Role of miRNA-182 and FOXO3 Expression in Breast Cancer. Asian Pac. J. Cancer Prev..

[142] Ma C., He D., Tian P., Wang Y., He Y., Wu Q., Jia Z., Zhang X., Zhang P., Ying H. (2022). miR-182 targeting reprograms tumor-associated macrophages and limits breast cancer progression. Proc. Natl. Acad. Sci. USA.

[143] Bašová P., Pešta M., Sochor M., Stopka T. (2017). Prediction potential of serum miR-155 and miR-24 for relapsing early breast cancer. Int. J. Mol. Sci..

[144] Roscigno G., Puoti I., Giordano I., Donnarumma E., Russo V., Affinito A., Adamo A., Quintavalle C., Todaro M., dM Vivanco M. (2017). MiR-24 induces chemotherapy resistance and hypoxic advantage in breast cancer. Oncotarget.

[145] Chen D., Fan Y., Wan F. (2020). LncRNA IGBP1-AS1/miR-24-1/ZIC3 loop regulates the proliferation and invasion ability in breast cancer. Cancer Cell Int..

[146] Zhao Z., Fan X., Jiang L., Xu Z., Xue L., Zhan Q., Song Y. (2017). miR-503-3p promotes epithelial–mesenchymal transition in breast cancer by directly targeting SMAD2 and E-cadherin. J. Genet. Genom..

[147] Dou D., Ren X., Han M., Xu X., Ge X., Gu Y., Wang X. (2020). Cancer-associated fibroblasts-derived exosomes suppress immune cell function in breast cancer via the miR-92/PD-L1 pathway. Front. Immunol..

[148] Wu H., Li S. (2020). Long non-coding RNA MT1JP exerts anti-cancer effects in breast cancer cells by regulating miR-92-3p. General. Physiol. Biophys..

[149] Garofalo M., Quintavalle C., Romano G., Croce C.M., Condorelli G. (2012). miR221/222 in cancer: Their role in tumor progression and response to therapy. Curr. Mol. Med..

[150] Piva R., Spandidos D.A., Gambari R. (2013). From microRNA functions to microRNA therapeutics: Novel targets and novel drugs in breast cancer research and treatment. Int. J. Oncol..

[151] Liang Y.-K., Lin H.-Y., Dou X.-W., Chen M., Wei X.-L., Zhang Y.-Q., Wu Y., Chen C.-F., Bai J.-W., Xiao Y.-S. (2018). MiR-221/222 promote epithelial-mesenchymal transition by targeting Notch3 in breast cancer cell lines. NPJ Breast Cancer.

[152] Ouyang Y.X., Feng J., Wang Z., Zhang G.J., Chen M. (2021). miR-221/222 sponge abrogates tamoxifen resistance in ER-positive breast cancer cells through restoring the expression of ERα. Mol. Biomed..

[153] Zhang L., Chen T., Yan L., Xu H., Wang Y., Li Y., Wang H., Chen S., Wang W., Chen C. (2019). MiR-155-3p acts as a tumor suppressor and reverses paclitaxel resistance via negative regulation of MYD88 in human breast cancer. Gene.

[154] Mattiske S., Suetani R.J., Neilsen P.M., Callen D.F. (2012). The Oncogenic Role of miR-155 in Breast CancermiR-155 and Breast Cancer. Cancer Epidemiol. Biomark. Prev..

[155] Jang M.H., Kim H.J., Gwak J.M., Chung Y.R., Park S.Y. (2017). Prognostic value of microRNA-9 and microRNA-155 expression in triple-negative breast cancer. Hum. Pathol..

[156] Shen S., Sun Q., Liang Z., Cui X., Ren X., Chen H., Zhang X., Zhou Y. (2014). A prognostic model of triple-negative breast cancer based on miR-27b-3p and node status. PLoS ONE.

[157] Shen S.-J., Song Y., Ren X.-Y., Xu Y.-L., Zhou Y.-D., Liang Z.-Y., Sun Q. (2020). MicroRNA-27b-3p promotes tumor progression and metastasis by inhibiting peroxisome proliferator-activated receptor gamma in triple-negative breast cancer. Front. Oncol..

[158] Wu Y., Shi W., Tang T., Wang Y., Yin X., Chen Y., Zhang Y., Xing Y., Shen Y., Xia T. (2019). miR-29a contributes to breast cancer cells epithelial–mesenchymal transition, migration, and invasion via down-regulating histone H4K20 trimethylation through directly targeting SUV420H2. Cell Death Dis..

[159] Yin L., Duan J.-J., Bian X.-W., Yu S.-C. (2020). Triple-negative breast cancer molecular subtyping and treatment progress. Breast Cancer Res..

[160] Fontana A., Barbano R., Dama E., Pasculli B., Rendina M., Morritti M.G., Melocchi V., Castelvetere M., Valori V.M., Ravaioli S. (2021). Combined analysis of miR-200 family and its significance for breast cancer. Sci. Rep..

[161] Le M.T., Hamar P., Guo C., Basar E., Perdigão-Henriques R., Balaj L., Lieberman J. (2014). miR-200–containing extracellular vesicles promote breast cancer cell metastasis. J. Clin. Investig..

[162] Cavallari I., Ciccarese F., Sharova E., Urso L., Raimondi V., Silic-Benussi M., D’Agostino D.M., Ciminale V. (2021). The miR-200 family of microRNAs: Fine tuners of epithelial-mesenchymal transition and circulating cancer biomarkers. Cancers.

[163] Simpson K.E., Watson K.L., Moorehead R.A. (2022). Elevated expression of miR-200c/141 in MDA-MB-231 cells suppresses MXRA8 levels and impairs breast cancer growth and metastasis in vivo. Genes.

[164] Li X.-Y., Luo Q.-F., Wei C.-K., Li D.-F., Li J., Fang L. (2014). MiRNA-107 inhibits proliferation and migration by targeting CDK8 in breast cancer. Int. J. Clin. Exp. Med..

[165] Luo Z., Zheng Y., Zhang W. (2018). Pleiotropic functions of miR107 in cancer networks. OncoTargets Ther..

[166] Kodahl A.R., Zeuthen P., Binder H., Knoop A.S., Ditzel H.J. (2014). Alterations in circulating miRNA levels following early-stage estrogen receptor-positive breast cancer resection in post-menopausal women. PLoS ONE.

[167] Ma L., Young J., Prabhala H., Pan E., Mestdagh P., Muth D., Teruya-Feldstein J., Reinhardt F., Onder T.T., Valastyan S. (2010). miR-9, a MYC/MYCN-activated microRNA, regulates E-cadherin and cancer metastasis. Nat. Cell Biol..

[168] Ewida H.A., Shabayek M., Seleem M. (2021). Evaluation of miRNAs 9 and 342 expressions in sera as diagnostic and prognostic biomarkers for breast cancer. Breast Dis..

[169] Bhardwaj A., Singh H., Rajapakshe K., Tachibana K., Ganesan N., Pan Y., Gunaratne P.H., Coarfa C., Bedrosian I. (2017). Regulation of miRNA-29c and its downstream pathways in preneoplastic progression of triple-negative breast cancer. Oncotarget.

[170] Li J., Lai Y., Ma J., Liu Y., Bi J., Zhang L., Chen L., Yao C., Lv W., Chang G. (2017). miR-17-5p suppresses cell proliferation and invasion by targeting ETV1 in triple-negative breast cancer. BMC Cancer.

[171] Xu X., Zhang Y., Jasper J., Lykken E., Alexander P.B., Markowitz G.J., McDonnell D.P., Li Q.-J., Wang X.-F. (2016). MiR-148a functions to suppress metastasis and serves as a prognostic indicator in triple-negative breast cancer. Oncotarget.

[172] Karthik L., Kumar G., Keswani T., Bhattacharyya A., Chandar S.S., Bhaskara Rao K. (2014). Protease inhibitors from marine actinobacteria as a potential source for antimalarial compound. PLoS ONE.

[173] Wang J., Li M., Han X., Wang H., Wang X., Ma G., Xia T., Wang S. (2020). MiR-1976 knockdown promotes epithelial–mesenchymal transition and cancer stem cell properties inducing triple-negative breast cancer metastasis. Cell Death Dis..

[174] Hao Y., Yang J., Yin S., Zhang H., Fan Y., Sun C., Gu J., Xi J.J. (2014). The synergistic regulation of VEGF-mediated angiogenesis through miR-190 and target genes. RNA.

[175] Chu H.W., Cheng C.W., Chou W.C., Hu L.Y., Wang H.W., Hsiung C.N., Hsu H.M., Wu P.E., Hou M.F., Shen C.Y. (2014). A novel estrogen receptor-microRNA 190a-PAR-1-pathway regulates breast cancer progression, a finding initially suggested by genome-wide analysis of loci associated with lymph-node metastasis. Hum. Mol. Genet..

[176] Krishnan K., Steptoe A.L., Martin H.C., Pattabiraman D.R., Nones K., Waddell N., Mariasegaram M., Simpson P.T., Lakhani S.R., Vlassov A. (2013). miR-139-5p is a regulator of metastatic pathways in breast cancer. RNA.

[177] Yan M., Li X., Tong D., Han C., Zhao R., He Y., Jin X. (2016). miR-136 suppresses tumor invasion and metastasis by targeting RASAL2 in triple-negative breast cancer. Oncol. Rep..

[178] García-Vazquez R., Ruiz-García E., Meneses García A., Astudillo-de la Vega H., Lara-Medina F., Alvarado-Miranda A., Maldonado-Martínez H., González-Barrios J.A., Campos-Parra A.D., Rodríguez Cuevas S. (2017). A microRNA signature associated with pathological complete response to novel neoadjuvant therapy regimen in triple-negative breast cancer. Tumor Biol..

[179] Zhao Z., Li L., Du P., Ma L., Zhang W., Zheng L., Lan B., Zhang B., Ma F., Xu B. (2019). Transcriptional downregulation of miR-4306 serves as a new therapeutic target for triple negative breast cancer. Theranostics.

[180] Chen Y., Huang S., Wu B., Fang J., Zhu M., Sun L., Zhang L., Zhang Y., Sun M., Guo L. (2017). Transforming growth factor-β1 promotes breast cancer metastasis by downregulating miR-196a-3p expression. Oncotarget.

[181] Abdallah R., Youness R., El Meckawy N., El Sebaaei A., Abdelmotaal A., Assal R. (2018). Crosstalk between hesperetin and miR-486-5p in triple-negative breast cancer (TNBC): An approach towards precision medicine. Ann. Oncol..

[182] Abdallah R., Youness R., El Meckawy N., El Sebaei A., Abdelmotaal A., Assal R. (2018). Paradoxical effects of miR-486-5p on the oncogenic and immunogenic profiles in triple negative breast cancer (TNBC). Eur. J. Cancer.

[183] Elkhouly A., Youness R., Gad M. (2019). miR-486-5p Counteracts the Shedding of MICA/B and CD155 Immune-Ligands in TNBC Patients. Ann. Oncol..

[184] Tang H., Liu P., Yang L., Xie X., Ye F., Wu M., Liu X., Chen B., Zhang L., Xie X. (2014). miR-185 Suppresses Tumor Proliferation by Directly Targeting E2F6 and DNMT1 and Indirectly Upregulating BRCA1 in Triple-Negative Breast Cancer. Mol. Cancer Ther..

[185] Hermeking H. (2010). The miR-34 family in cancer and apoptosis. Cell Death Differ..

[186] Maroof H., Salajegheh A., Anthony Smith R., King-Yin Lam A. (2014). MicroRNA-34 family, mechanisms of action in cancer: A review. Curr. Cancer Drug Targets.

[187] Misso G., Di Martino M.T., De Rosa G., Farooqi A.A., Lombardi A., Campani V., Zarone M.R., Gullà A., Tagliaferri P., Tassone P. (2014). Mir-34: A new weapon against cancer?. Mol. Ther.-Nucleic Acids.

[188] Nie D., Fu J., Chen H., Cheng J., Fu J. (2019). Roles of microRNA-34a in epithelial to mesenchymal transition, competing endogenous RNA sponging and its therapeutic potential. Int. J. Mol. Sci..

[189] Umeh-Garcia M., Simion C., Ho P.-Y., Batra N., Berg A.L., Carraway K.L., Yu A., Sweeney C. (2020). A Novel Bioengineered miR-127 Prodrug Suppresses the Growth and Metastatic Potential of Triple-Negative Breast Cancer Cells. Cancer Res..

[190] Hu J., Xu J., Wu Y., Chen Q., Zheng W., Lu X., Zhou C., Jiao D. (2015). Identification of microRNA-93 as a functional dysregulated miRNA in triple-negative breast cancer. Tumor Biol..

[191] Yang M., Xiao R., Wang X., Xiong Y., Duan Z., Li D., Kan Q. (2022). MiR-93-5p regulates tumorigenesis and tumor immunity by targeting PD-L1/CCND1 in breast cancer. Ann. Transl. Med..

[192] Bao C., Chen J., Chen D., Lu Y., Lou W., Ding B., Xu L., Fan W. (2020). MiR-93 suppresses tumorigenesis and enhances chemosensitivity of breast cancer via dual targeting E2F1 and CCND1. Cell Death Dis..

[193] Cai W.-L., Huang W.-D., Li B., Chen T.-R., Li Z.-X., Zhao C.-L., Li H.-Y., Wu Y.-M., Yan W.-J., Xiao J.-R. (2018). microRNA-124 inhibits bone metastasis of breast cancer by repressing Interleukin-11. Mol. Cancer.

[194] Miao Y., Lu J., Fan B., Sun L. (2020). MicroRNA-126-5p inhibits the migration of breast cancer cells by directly targeting CNOT7. Technol. Cancer Res. Treat..

[195] Alhasan L. (2019). MiR-126 modulates angiogenesis in breast cancer by targeting VEGF-A-mRNA. Asian Pac. J. Cancer Prev. APJCP.

[196] Hong Z., Hong C., Ma B., Wang Q., Zhang X., Li L., Wang C., Chen D. (2019). MicroRNA-126-3p inhibits the proliferation, migration, invasion, and angiogenesis of triple-negative breast cancer cells by targeting RGS3. Oncol. Rep..

[197] Msheik Z.S., Nassar F.J., Chamandi G., Itani A.R., Gadaleta E., Chalala C., Alwan N., Nasr R.R. (2022). miR-126 Decreases Proliferation and Mammosphere Formation of MCF-7 and Predicts Prognosis of ER+ Breast Cancer. Diagnostics.

[198] Zhang G., Wang J., Zheng R., Song B., Huang L., Liu Y., Hao Y., Bai X. (2020). MiR-133 targets YES1 and inhibits the growth of triple-negative breast cancer cells. Technol. Cancer Res. Treat..

[199] Ramaiah M.J., Lavanya A., Honarpisheh M., Zarea M., Bhadra U., Bhadra M.P. (2014). miR-15/16 complex targets p70S6 kinase1 and controls cell proliferation in MDA-MB-231 breast cancer cells. Gene.

[200] Srinivas C., Ramaiah M.J., Lavanya A., Yerramsetty S., Kavi Kishor P., Basha S.A., Kamal A., Bhadra U., Bhadra M.-P. (2015). Novel etoposide analogue modulates expression of angiogenesis associated microRNAs and regulates cell proliferation by targeting STAT3 in breast cancer. PLoS ONE.

[201] Li P., Dong J., Zhou X., Sun W., Huang H., Chen T., Ye B., Zheng Z., Lu M. (2017). Expression patterns of microRNA-329 and its clinical performance in diagnosis and prognosis of breast cancer. OncoTargets Ther..

[202] Ali Ahmed E., Abd El-Basit S.A., Mohamed M.A., Swellam M. (2022). Clinical role of MiRNA 29a and MiRNA 335 on breast cancer management: Their relevance to MMP2 protein level. Arch. Physiol. Biochem..

[203] Liu X., Wang J., Zhang G. (2019). miR-4458 regulates cell proliferation and apoptosis through targeting SOCS1 in triple-negative breast cancer. J. Cell. Biochem..

[204] Wong C.K., Gromisch C., Ozturk S., Papageorgis P., Abdolmaleky H.M., Reinhard B.M., Thiagalingam A., Thiagalingam S. (2019). MicroRNA-4417 is a tumor suppressor and prognostic biomarker for triple-negative breast cancer. Cancer Biol. Ther..

[205] Yin K., Yin W., Wang Y., Zhou L., Liu Y., Yang G., Wang J., Lu J. (2016). MiR-206 suppresses epithelial mesenchymal transition by targeting TGF-β signaling in estrogen receptor positive breast cancer cells. Oncotarget.

[206] Samaeekia R., Adorno-Cruz V., Bockhorn J., Chang Y.-F., Huang S., Prat A., Ha N., Kibria G., Huo D., Zheng H. (2017). miR-206 inhibits stemness and metastasis of breast cancer by targeting MKL1/IL11 Pathwaymir-206 inhibits stemness and metastasis. Clin. Cancer Res..

[207] Zhou Y., Wang M., Tong Y., Liu X., Zhang L., Dong D., Shao J., Zhou Y. (2019). miR-206 promotes cancer progression by targeting full-length neurokinin-1 receptor in breast cancer. Technol. Cancer Res. Treat..

[208] Gharib A.F., Khalifa A.S., Eed E.M., Banjer H.J., Shami A.A., El Askary A., Elsawy W.H. (2022). Role of MicroRNA-31 (miR-31) in Breast Carcinoma Diagnosis and Prognosis. In Vivo.

[209] Tang L., Chen Y., Tang X., Wei D., Xu X., Yan F. (2020). Long Noncoding RNA <i>DCST1-AS1</i> Promotes Cell Proliferation and Metastasis in Triple-negative Breast Cancer by Forming a Positive Regulatory Loop with miR-873-5p and MYC. J. Cancer.

[210] Yao M., Wang S., Chen L., Wei B., Fu P. (2022). Research on correlations of miR-585 expression with progression and prognosis of triple-negative breast cancer. Clin. Exp. Med..

[211] Liu B., Pan J., Fu C. (2021). Correlation of microRNA-367 in the clinicopathologic features and prognosis of breast cancer patients. Medicine.

[212] Terkelsen T., Russo F., Gromov P., Haakensen V.D., Brunak S., Gromova I., Krogh A., Papaleo E. (2020). Secreted breast tumor interstitial fluid microRNAs and their target genes are associated with triple-negative breast cancer, tumor grade, and immune infiltration. Breast Cancer Res..

[213] Kalinina T.S., Kononchuk V.V., Yakovleva A.K., Alekseenok E.Y., Sidorov S.V., Gulyaeva L.F. (2020). Association between lymph node status and expression levels of androgen receptor, miR-185, miR-205, and miR-21 in breast cancer subtypes. Int. J. Breast Cancer.

[214] Lin L.-F., Li Y.-T., Han H., Lin S.-G. (2021). MicroRNA-205-5p targets the HOXD9-Snail1 axis to inhibit triple negative breast cancer cell proliferation and chemoresistance. Aging.

[215] Plantamura I., Cataldo A., Cosentino G., Iorio M.V. (2020). miR-205 in breast cancer: State of the art. Int. J. Mol. Sci..

[216] Shen Y., Xu Y., Huang L., Chi Y., Meng L. (2021). MiR-205 suppressed the malignant behaviors of breast cancer cells by targeting CLDN11 via modulation of the epithelial-to-mesenchymal transition. Aging.

[217] Tokumaru Y., Oshi M., Patel A., Katsuta E., Yan L., Angarita F.A., Dasgupta S., Nagahashi M., Matsuhashi N., Futamura M. (2021). Low expression of miR-195 is associated with cell proliferation, glycolysis and poor survival in estrogen receptor (ER)-positive but not in triple negative breast cancer. Am. J. Cancer Res..

[218] McAnena P., Tanriverdi K., Curran C., Gilligan K., Freedman J.E., Brown J.A., Kerin M.J. (2019). Circulating microRNAs miR-331 and miR-195 differentiate local luminal a from metastatic breast cancer. BMC Cancer.

[219] Wang L., Kang F.-b., Wang J., Yang C., He D.-w. (2019). Downregulation of miR-205 contributes to epithelial–mesenchymal transition and invasion in triple-negative breast cancer by targeting HMGB1–RAGE signaling pathway. Anti-Cancer Drugs.

[220] Thammaiah C.K., Jayaram S. (2016). Role of let-7 family microRNA in breast cancer. Non-Coding RNA Res..

[221] Yan Y., Zhang F., Fan Q., Li X., Zhou K. (2014). Breast cancer-specific TRAIL expression mediated by miRNA response elements of let-7 and miR-122. Neoplasma.

[222] Jiang R., Li Y., Zhang A., Wang B., Xu Y., Xu W., Zhao Y., Luo F., Liu Q. (2014). The acquisition of cancer stem cell-like properties and neoplastic transformation of human keratinocytes induced by arsenite involves epigenetic silencing of let-7c via Ras/NF-κB. Toxicol. Lett..

[223] Zhu L., Zhang Y.-J., Wang B., Yang L., Zheng Y.-Q., Sun L.-D., Tian L., Chen T., Wang J.-D. (2021). PCDHB17P/miR-145-3p/MELK/NF-κB feedback loop promotes metastasis and angiogenesis of breast cancer. Front. Oncol..

[224] Tang W., Zhang X., Tan W., Gao J., Pan L., Ye X., Chen L., Zheng W. (2019). miR-145-5p suppresses breast cancer progression by inhibiting SOX2. J. Surg. Res..

[225] Itani M.M., Nassar F.J., Tfayli A.H., Talhouk R.S., Chamandi G.K., Itani A.R.S., Makoukji J., Boustany R.-M.N., Hou L., Zgheib N.K. (2021). A signature of four circulating microRNAs as potential biomarkers for diagnosing early-stage breast cancer. Int. J. Mol. Sci..

[226] Zheng M., Wu Z., Wu A., Huang Z., He N., Xie X. (2016). MiR-145 promotes TNF-α-induced apoptosis by facilitating the formation of RIP1-FADDcaspase-8 complex in triple-negative breast cancer. Tumor Biol..

[227] Qian J., Lei X., Sun Y., Zheng L., Li J., Zhang S., Zhang L., Li W., Shi J., Jia W. (2021). Long non-coding RNA SNHG8 enhances triple-negative breast cancer cell proliferation and migration by regulating the miR-335-5p/PYGO2 axis. Biol. Direct.

[228] Chen T., Dong Y., Wu X. (2022). Plasma exosomal miR-335-5p serves as a diagnostic indicator and inhibits immune escape in triple-negative breast cancer. Xi Bao Yu Fen Zi Mian Yi Xue Za Zhi Chin. J. Cell. Mol. Immunol..

[229] Hao J., Lai M., Liu C. (2019). Expression of miR-335 in triple-negative breast cancer and its effect on chemosensitivity. J. Buon.

[230] Cao D., Zhu H., Zhao Q., Huang J., Zhou C., He J., Liang Y. (2020). MiR-128 suppresses metastatic capacity by targeting metadherin in breast cancer cells. Biol. Res..

[231] Xiao M., Lou C., Xiao H., Yang Y., Cai X., Li C., Jia S., Huang Y. (2018). MiR-128 regulation of glucose metabolism and cell proliferation in triple-negative breast cancer. J. Br. Surg..

[232] Kodahl A.R., Lyng M.B., Binder H., Cold S., Gravgaard K., Knoop A.S., Ditzel H.J. (2014). Novel circulating microRNA signature as a potential non-invasive multi-marker test in ER-positive early-stage breast cancer: A case control study. Mol. Oncol..

[233] Gong J., Luk F., Jaiswal R., Bebawy M. (2014). Microparticles mediate the intercellular regulation of microRNA-503 and proline-rich tyrosine kinase 2 to alter the migration and invasion capacity of breast cancer cells. Front. Oncol..

[234] Li Y., Li W., Ying Z., Tian H., Zhu X., Li J., Li M. (2014). Metastatic Heterogeneity of Breast Cancer Cells Is Associated with Expression of a Heterogeneous TGFβ-Activating miR424–503 Gene ClustermiR424–503 Activates TGFβ and Promotes Breast Cancer Metastasis. Cancer Res..

[235] Rodriguez-Barrueco R., Nekritz E.A., Bertucci F., Yu J., Sanchez-Garcia F., Zeleke T.Z., Gorbatenko A., Birnbaum D., Ezhkova E., Cordon-Cardo C. (2017). miR-424 (322)/503 is a breast cancer tumor suppressor whose loss promotes resistance to chemotherapy. Genes Dev..

[236] Long J., Ou C., Xia H., Zhu Y., Liu D. (2015). MiR-503 inhibited cell proliferation of human breast cancer cells by suppressing CCND1 expression. Tumor Biol..

[237] Martinez-Useros J., Martin-Galan M., Florez-Cespedes M., Garcia-Foncillas J. (2021). Epigenetics of Most Aggressive Solid Tumors: Pathways, Targets and Treatments. Cancers.

[238] Qayoom H., Wani N.A., Alshehri B., Mir M.A. (2021). An insight into the cancer stem cell survival pathways involved in chemoresistance in triple-negative breast cancer. Future Oncol..

[239] Kudelova E., Smolar M., Holubekova V., Hornakova A., Dvorska D., Lucansky V., Koklesova L., Kudela E., Kubatka P. (2022). Genetic Heterogeneity, Tumor Microenvironment and Immunotherapy in Triple-Negative Breast Cancer. Int. J. Mol. Sci..

[240] Kwong S.C., Jamil A.H.A., Rhodes A., Taib N.A., Chung I. (2019). Metabolic role of fatty acid binding protein 7 in mediating triple-negative breast cancer cell death via PPAR-α signaling. J. Lipid Res..

[241] Tang A.H., Hoefer R.A., Guye M.L., Bear H.D. (2022). Persistent EGFR/K-RAS/SIAH pathway activation drives chemo-resistance and early tumor relapse in triple-negative breast cancer. Cancer Drug Resist..

[242] Spina A., Di Maiolo F., Esposito A., D’Auria R., Di Gesto D., Chiosi E., Sorvillo L., Naviglio S. (2013). Integrating leptin and cAMP signalling pathways in triple-negative breast cancer cells. Front. Biosci. (Landmark Ed.).

[243] Evans K.W., Yuca E., Scott S.S., Zhao M., Paez Arango N., Cruz Pico C.X., Saridogan T., Shariati M., Class C.A., Bristow C.A. (2021). Oxidative Phosphorylation Is a Metabolic Vulnerability in Chemotherapy-Resistant Triple-Negative Breast Cancer. Cancer Res..

[244] Heeke A.L., Tan A.R. (2021). Checkpoint inhibitor therapy for metastatic triple-negative breast cancer. Cancer Metastasis Rev..

[245] Qu C., Peng Y., Liu S. (2022). Ferroptosis Biology and Implication in Cancers. Front. Mol. Biosci..

[246] Shan C., Liang Y., Wang K., Li P. (2023). Noncoding RNAs in cancer ferroptosis: From biology to clinical opportunity. Biomed. Pharmacother..

[247] Liu Y., Hu Y., Jiang Y., Bu J., Gu X. (2022). Targeting ferroptosis, the achilles’ heel of breast cancer: A review. Front. Pharmacol..

[248] Culig Z., Santer F.R. (2014). Androgen receptor signaling in prostate cancer. Cancer Metastasis Rev..

[249] Waltering K.K., Porkka K.P., Jalava S.E., Urbanucci A., Kohonen P.J., Latonen L.M., Kallioniemi O.P., Jenster G., Visakorpi T. (2011). Androgen regulation of micro-RNAs in prostate cancer. Prostate.

[250] Fletcher C.E., Dart D.A., Sita-Lumsden A., Cheng H., Rennie P.S., Bevan C.L. (2012). Androgen-regulated processing of the oncomir miR-27a, which targets Prohibitin in prostate cancer. Hum. Mol. Genet..

[251] Nilsson E.M., Laursen K.B., Whitchurch J., McWilliam A., Ødum N., Persson J.L., Heery D.M., Gudas L.J., Mongan N.P. (2015). MiR137 is an androgen regulated repressor of an extended network of transcriptional coregulators. Oncotarget.

[252] Brinkmann A.O., Faber P.W., van Rooij H.C., Kuiper G.G., Ris C., Klaassen P., van der Korput J.A., Voorhorst M.M., van Laar J.H., Mulder E. (1989). The human androgen receptor: Domain structure, genomic organization and regulation of expression. J. Steroid Biochem..

[253] Kuiper G.G., Faber P.W., van Rooij H.C., van der Korput J.A., Ris-Stalpers C., Klaassen P., Trapman J., Brinkmann A.O. (1989). Structural organization of the human androgen receptor gene. J. Mol. Endocrinol..

[254] Brinkmann A.O. (2001). Molecular basis of androgen insensitivity. Mol. Cell Endocrinol..

[255] Burger H.G. (2002). Androgen production in women. Fertil. Steril..

[256] McNamara K.M., Moore N.L., Hickey T.E., Sasano H., Tilley W.D. (2014). Complexities of androgen receptor signalling in breast cancer. Endocr. Relat. Cancer.

[257] Shi G.M., Xu Y., Fan J., Zhou J., Yang X.R., Qiu S.J., Liao Y., Wu W.Z., Ji Y., Ke A.W. (2008). Identification of side population cells in human hepatocellular carcinoma cell lines with stepwise metastatic potentials. J. Cancer Res. Clin. Oncol..

[258] Al-Othman N., Ahram M., Alqaraleh M. (2020). Role of androgen and microRNA in triple-negative breast cancer. Breast Dis..

[259] Xu S., Wang T., Song W., Jiang T., Zhang F., Yin Y., Jiang S.-W., Wu K., Yu Z., Wang C. (2015). The inhibitory effects of AR/miR-190a/YB-1 negative feedback loop on prostate cancer and underlying mechanism. Sci. Rep..

[260] Wang D.Y., Allen D.S., De Stavola B.L., Fentiman I.S., Brussen J., Bulbrook R.D., Thomas B.S., Hayward J.L., Reed M.J. (2000). Urinary androgens and breast cancer risk: Results from a long-term prospective study based in Guernsey. Br. J. Cancer.

[261] Loibl S., Müller B.M., von Minckwitz G., Schwabe M., Roller M., Darb-Esfahani S., Ataseven B., du Bois A., Fissler-Eckhoff A., Gerber B. (2011). Androgen receptor expression in primary breast cancer and its predictive and prognostic value in patients treated with neoadjuvant chemotherapy. Breast Cancer Res. Treat..

[262] Tang D., Xu S., Zhang Q., Zhao W. (2012). The expression and clinical significance of the androgen receptor and E-cadherin in triple-negative breast cancer. Med. Oncol..

[263] Shi Y., Yang F., Sun Z., Zhang W., Gu J., Guan X. (2017). Differential microRNA expression is associated with androgen receptor expression in breast cancer. Mol. Med. Rep..

[264] Yan L.X., Wu Q.N., Zhang Y., Li Y.Y., Liao D.Z., Hou J.H., Fu J., Zeng M.S., Yun J.P., Wu Q.L. (2011). Knockdown of miR-21 in human breast cancer cell lines inhibits proliferation, in vitro migration and in vivotumor growth. Breast Cancer Res..

[265] Guan C., Zhang L., Wang S., Long L., Zhou H., Qian S., Ma M., Bai F., Meng Q.H., Lyu J. (2019). Upregulation of MicroRNA-21 promotes tumorigenesis of prostate cancer cells by targeting KLF5. Cancer Biol. Ther..

[266] Gui B., Hsieh C.-L., Kantoff P.W., Kibel A.S., Jia L. (2017). Androgen receptor-mediated downregulation of microRNA-221 and-222 in castration-resistant prostate cancer. PLoS ONE.

[267] Ichikawa T., Sato F., Terasawa K., Tsuchiya S., Toi M., Tsujimoto G., Shimizu K. (2012). Trastuzumab produces therapeutic actions by upregulating miR-26a and miR-30b in breast cancer cells. PLoS ONE.

[268] Bandini E., Fanini F., Vannini I., Rossi T., Plousiou M., Tumedei M.M., Limarzi F., Maltoni R., Fabbri F., Hrelia S. (2020). miR-9-5p as a regulator of the androgen receptor pathway in breast cancer cell lines. Front. Cell Dev. Biol..

[269] Al-Othman N., Hammad H., Ahram M. (2018). Dihydrotestosterone regulates expression of CD44 via miR-328-3p in triple-negative breast cancer cells. Gene.

[270] Aakula A., Leivonen S.-K., Hintsanen P., Aittokallio T., Ceder Y., Børresen-Dale A.-L., Perälä M., Östling P., Kallioniemi O. (2015). MicroRNA-135b regulates ERα, AR and HIF1AN and affects breast and prostate cancer cell growth. Mol. Oncol..

[271] Guo Q., Qiu P., Yao Q., Chen J., Lin J. (2022). Integrated Bioinformatics Analysis for the Screening of Hub Genes and Therapeutic Drugs in Androgen Receptor-Positive TNBC. Dis. Markers.

[272] Qiu P., Guo Q., Yao Q., Chen J., Lin J. (2021). Hsa-mir-3163 and CCNB1 may be potential biomarkers and therapeutic targets for androgen receptor positive triple-negative breast cancer. PLoS ONE.

[273] Bandini E., Fanini F. (2019). MicroRNAs and Androgen Receptor: Emerging Players in Breast Cancer. Front. Genet..

[274] Lyu S., Yu Q., Ying G., Wang S., Wang Y., Zhang J., Niu Y. (2014). Androgen receptor decreases CMYC and KRAS expression by upregulating let-7a expression in ER−, PR−, AR+ breast cancer. Int. J. Oncol..

[275] Zhang W., Liu X., Liu S., Qin Y., Tian X., Niu F., Liu H., Liu N., Niu Y. (2018). Androgen receptor/let-7a signaling regulates breast tumor-initiating cells. Oncotarget.

[276] Nadiminty N., Tummala R., Lou W., Zhu Y., Zhang J., Chen X., White R.W.d., Kung H.-J., Evans C.P., Gao A.C. (2012). MicroRNA let-7c suppresses androgen receptor expression and activity via regulation of Myc expression in prostate cancer cells. J. Biol. Chem..

[277] Lyu S., Liu H., Liu X., Liu S., Wang Y., Yu Q., Niu Y. (2017). Interrelation of androgen receptor and miR-30a and miR-30a function in ER(−), PR(−), AR(+) MDA-MB-453 breast cancer cells. Oncol. Lett..

[278] Ahram M., Mustafa E., Zaza R., Abu Hammad S., Alhudhud M., Bawadi R., Zihlif M. (2017). Differential expression and androgen regulation of microRNAs and metalloprotease 13 in breast cancer cells. Cell Biol. Int..

[279] Xie H., Xiao R., He Y., He L., Xie C., Chen J., Hong Y. (2021). MicroRNA-100 inhibits breast cancer cell proliferation, invasion and migration by targeting FOXA1. Oncol. Lett..

[280] Yang F., Shen Y., Zhang W., Jin J., Huang D., Fang H., Ji W., Shi Y., Tang L., Chen W. (2018). An androgen receptor negatively induced long non-coding RNA ARNILA binding to miR-204 promotes the invasion and metastasis of triple-negative breast cancer. Cell Death Differ..

[281] Nakano K., Miki Y., Hata S., Ebata A., Takagi K., Mcnamara K.M., Sakurai M., Masuda M., Hirakawa H., Ishida T. (2013). Identification of androgen-responsive microRNAs and androgen-related genes in breast cancer. Anticancer Res..

[282] Guo J., Mei Y., Li K., Huang X., Yang H. (2016). Downregulation of miR-17-92a cluster promotes autophagy induction in response to celastrol treatment in prostate cancer cells. Biochem. Biophys. Res. Commun..

[283] Wang S., Yang Y., Cao Y.-D., Tang X.-X., Du P. (2021). Androgen downregulation of miR-760 promotes prostate cancer cell growth by regulating IL6. Asian J. Androl..

[284] Naidoo M., Levine F., Gillot T., Orunmuyi A.T., Olapade-Olaopa E.O., Ali T., Krampis K., Pan C., Dorsaint P., Sboner A. (2021). MicroRNA-1205 Regulation of FRYL in Prostate Cancer. Front. Cell Dev. Biol..

[285] Rönnau C., Fussek S., Smit F., Aalders T., van Hooij O., Pinto P., Burchardt M., Schalken J., Verhaegh G. (2021). Upregulation of miR-3195, miR-3687 and miR-4417 is associated with castration-resistant prostate cancer. World J. Urol..

[286] Martínez-González L.J., Sánchez-Conde V., González-Cabezuelo J.M., Antunez-Rodríguez A., Andrés-León E., Robles-Fernandez I., Lorente J.A., Vázquez-Alonso F., Alvarez-Cubero M.J. (2021). Identification of MicroRNAs as viable aggressiveness biomarkers for prostate cancer. Biomedicines.

